# Effizientere Ressourcennutzung in Forschungslaboratorien durch Qualitätsmanagement

**DOI:** 10.1007/s00103-025-04074-w

**Published:** 2025-06-05

**Authors:** Sophia Sohns, Janine Kleymann-Hilmes

**Affiliations:** Forschungskoordination, Robert Koch-Institut, Berlin, Deutschland

**Keywords:** Laboratorien, Nachhaltigkeit, Gute wissenschaftliche Praxis, Qualitätssicherung, Wissenschaft, Laboratories, Sustainability, Good scientific practice, Quality assurance, Science

## Abstract

Forschung und Qualitätsmanagement (QM) könnten auf den ersten Blick kaum unterschiedlicher sein. Forschung ist geprägt von Freiheit, Kreativität und Flexibilität. QM hingegen erscheint oft starr, strikt und bürokratisch. Dennoch können Forschungslabore erheblich von den Prinzipien des QM profitieren, insbesondere wenn es darum geht, die Qualität und Effizienz der Forschung zu steigern und damit die stets limitierten Ressourcen bestmöglich einzusetzen.

Eine hohe Forschungsqualität hängt von verschiedenen Faktoren ab. Diese unterschiedlichen Einflussfaktoren wurden detailliert betrachtet und in einem spezifischen Forschungsqualitätsmanagementsystem in Pilotlaboratorien des Robert Koch-Instituts (RKI) und an einer universitären Einrichtung umgesetzt. Das Forschungsqualitätsmanagementsystem basiert dabei auf den Anforderungen der guten wissenschaftlichen Praxis. Es zeigte sich, dass viele Labore bereits Prozesse etabliert haben, die durch ein Qualitätsmanagementsystem (QMS) weiter optimiert und effizienter gestaltet werden können.

Zur Bewertung des Reifegrads der Laborprozesse wurde ein Selbstbewertungstool für Laborleitende entwickelt. Dieser Bericht bietet einen Überblick über die Qualität in der Forschung und zeigt Wege auf, wie diese auf Arbeitsgruppenebene individuell auf ihre Rahmenbedingungen abgestimmt verbessert werden kann. Dazu wird ein QMS vorgeschlagen und ein Werkzeug zur Selbstbewertung der eigenen Prozesse vorgestellt. Der Bericht stützt sich auf eine umfassende Literaturrecherche sowie auf ein abteilungs- und institutsübergreifendes Projekt des RKI.

**Zusatzmaterial online:**

Zusätzliche Informationen sind in der Online-Version dieses Artikels (10.1007/s00103-025-04074-w) enthalten.

## Abstract (en)

At first glance, research and quality management (QM) could hardly be more different. Research is characterized by freedom, creativity, and flexibility. QM often appears rigid, strict, and bureaucratic. Nevertheless, research laboratories can benefit significantly from the principles of QM, especially when it comes to increasing the quality and efficiency of research and thus making the best possible use of resources that are constantly limited.

High research quality depends on various factors. These different influencing factors were examined in detail and implemented in a specific research quality management system (QMS) in pilot laboratories of the Robert Koch Institute (RKI) and at a university institution. The research QMS is based on the requirements of good scientific practice. It was shown that many laboratories have already established processes that can be further optimized and made more efficient by a QMS.

A self-assessment tool for laboratory managers was developed to assess the maturity level of laboratory processes. This report provides an overview of the quality in research and shows ways in which this can be improved at the level of the working groups, tailored to their individual framework conditions. For this purpose, a QMS is proposed and a tool for the self-assessment of one’s own processes is presented. The report is based on a comprehensive literature review and a cross-departmental and cross-institutional project of the RKI.

## Einleitung

Forschung lebt von Freiheit – der Freiheit, neue Forschungsfragen zu identifizieren und diese durch kreative Ansätze zu lösen. Auch Serendipität, also die Entdeckung von Neuem durch unerwartete Beobachtungen oder vermeintliche Fehler, ist ein charakteristisches Merkmal der Forschung. Sie hat zu bahnbrechenden Entdeckungen wie der des Penicillins [1] oder der Röntgenstrahlung [2] geführt. Demgegenüber stehen die Prinzipien des Qualitätsmanagements (QM). QM, häufig mit Bürokratie, hohem Zeitaufwand und Starrheit gleichgesetzt, ist in der Forschung bislang selten anzutreffen. Kritiker befürchten, dass es den Forschungsgeist und die Kreativität einschränke und sich nur schwer mit wechselnden Fragestellungen und experimentellen Forschungsansätzen vereinbaren lasse [3]. Hinzu kommen kurze Förderperioden, begrenzte Ressourcen und fehlende gesetzliche Vorgaben – anders als in medizinischen oder Prüflaboren besteht weder eine gesetzliche Pflicht noch ein marktbasierter Druck, ein QM-System (QMS) einzuführen.

Dennoch erkennen einige Forschende im QM eine Chance für die Einrichtungen [3–6]. QM bietet Potenzial zur Optimierung von Laborprozessen, zur Steigerung der Forschungsqualität und zur effizienten Nutzung begrenzter Ressourcen [7]. Das Robert Koch-Institut (RKI) hat ein spezifisches QMS für Forschungslabore entwickelt und in Pilotlaboren sowohl im eigenen Haus als auch bei externen Kooperationspartnern eingeführt. Die Analyse der vielfältigen Forschungsprozesse in den beteiligten Teams zeigte sowohl Gemeinsamkeiten als auch Unterschiede, die wiederrum Stärken und Schwächen bestehender Strukturen aufzeigten.

## Forschungspraxis

Forschungslabore sind nicht völlig frei in ihrem Handeln, sondern unterliegen verschiedenen Rahmenbedingungen. Dazu zählen gesetzliche und behördliche Vorgaben, Anforderungen von Fördergesellschaften sowie Kodizes für gutes wissenschaftliches Arbeiten. Beispiele hierfür sind die „gute wissenschaftliche Praxis“ (GWP; [8]) der Deutschen Forschungsgemeinschaft (DFG) oder der „European Code of Conduct“ [9] der All European Academies. Diese Richtlinien stellen Anforderungen an Forschende und ihre Arbeitsweise mit dem Ziel, eine Kultur der wissenschaftlichen Integrität zu fördern [10]. Sie definieren das Berufsethos und dienen als Verhaltenskodex. Ihre Einhaltung ist essenziell für die Qualität der Forschung und die damit verbundene wissenschaftliche Karriere.

**Abb. 1:**
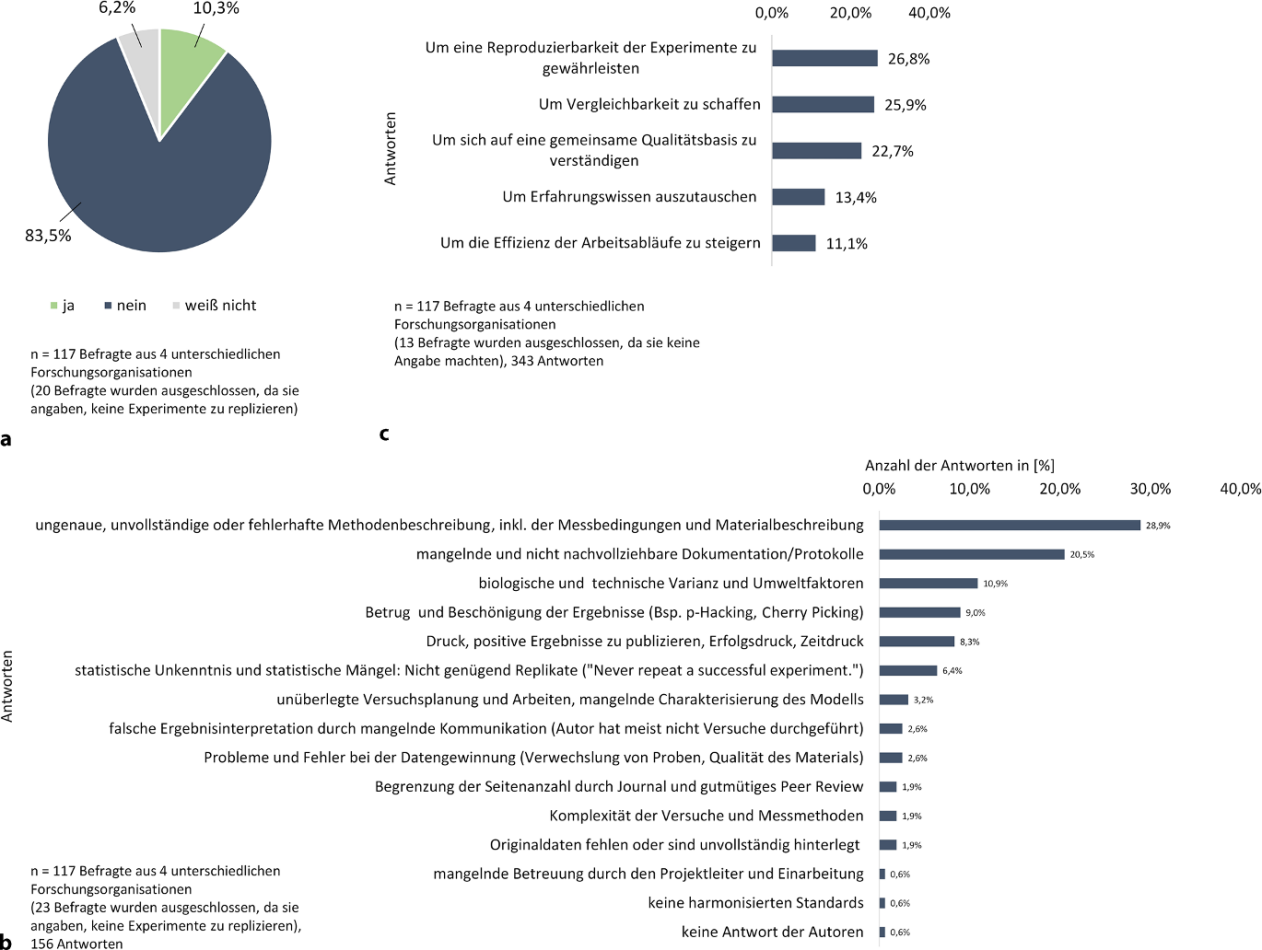
Ergebnis der Mitarbeiterbefragung des Robert Koch-Insituts (RKI) in unterschiedlichen Organisationen; **a** „Gelingt es Ihnen immer, eigene oder fremde Experimente zu replizieren?“ **b** „Welche Faktoren führen Ihrer Meinung nach zu einer geringen Replizierbarkeit in der biomedizinischen Forschung?“ **c** „Sind Sie der Meinung, dass gemeinsame Qualitätsstandards in der Forschung einen positiven Impact auf die biomedizinische Forschung haben könnten? Wenn ja, warum?“

Trotz dieser Rahmenbedingungen gibt es Fehlentwicklungen. Die Zahl der Widerrufe von Veröffentlichungen nimmt stetig zu [11] und die Reproduzierbarkeit von Experimenten ist oft unzureichend [12]. Dies bestätigt eine anonyme Mitarbeiterbefragung des RKI aus dem Jahr 2024, die in verschiedenen internen und externen Forschungslaboren durchgeführt wurde. Die in der Zeitschrift *Nature* [12] veröffentlichten Umfrageergebnisse sind weiterhin relevant, wie Abb. 1a zeigt: 83,5 % der Befragten berichteten, dass sie Schwierigkeiten hatten, Experimente zu reproduzieren.

Die häufigsten Ursachen wurden in ungenauen, unvollständigen oder fehlerhaften Methoden- und Materialbeschreibungen sowie in unzureichend dokumentierten Messbedingungen gesehen. Weitere Gründe waren lückenhafte Protokolle und Dokumentationen (Abb. 1b). Gleichzeitig zeigte die Befragung, dass die Mehrheit der Mitarbeitenden (84,5 %, *n* = 117, 1 ohne Antwort) von den potenziellen positiven Effekten gemeinsamer Qualitätsstandards überzeugt ist. Sie sehen darin die Chance, Reproduzierbarkeit und Vergleichbarkeit zu verbessern sowie ein einheitliches Qualitätsverständnis in der biomedizinischen Forschung zu fördern (Abb. 1c).

Um hier anzusetzen, ist es notwendig, die Forschungspraxis verschiedener Labore genauer zu betrachten. Forschenden dürften die folgenden Szenarien aus dem Forschungsalltag bekannt vorkommen – sei es aus dem eigenen Labor oder durch Erfahrungen in anderen:

- Umgang mit begrenzten Ressourcen,
- häufig wechselnde Teammitglieder, u. a. durch befristete Arbeitsverträge,
- Nutzung von Multi-User-Geräten ohne klare Angaben zu Zustand, Kalibrierung oder Wartung,
- unbekannte Proben in Geräten, deren Besitzer nicht auffindbar ist,
- uneinheitlich geführte Laborbücher, die sich in Struktur und Detailtiefe stark unterscheiden,
- zeitaufwendige Fehlersuchen.

Der Erfolg der Forschung hängt wesentlich von der Qualität ab. Wie die Forschungs- und Unterstützungsprozesse organisiert sind, variiert jedoch stark von Team zu Team. Einige Labore scheinen sich an dem Zitat von Albert Einstein zu orientieren: „Ordnung braucht nur der Dumme, das Genie beherrscht das Chaos.“ Leider ist Chaos selten effizient – insbesondere angesichts hoher Mitarbeiterfluktuation und kurzer Förderperioden.

Ein erweitertes Qualitätsverständnis über die GWP hinaus sowie ein Bewusstsein für die eigene Laborqualität und deren gezielte Verbesserung können Abhilfe schaffen.

## Qualität in der Forschung

Dafür ist die Klärung des Begriffs „Qualität“ notwendig. International wird sie definiert als der „Grad, in dem ein Satz inhärenter Merkmale eines Objektes Anforderungen erfüllt“ [13]. Anders gesagt: Qualität wird durch den Kunden und die Erfüllung seiner Anforderungen an die dauerhaften Merkmale eines Produkts bestimmt. Diese abstrakte Definition lässt sich auch auf die biomedizinische Forschung und deren Qualität übertragen. „Kunden“ der Forschung sind Stakeholder, die das „Produkt“, nämlich das neu generierte Wissen empfangen und weiterverwenden, also z. B. andere Forschende, die Industrie oder die Politik. Diese „Kunden“ stellen Anforderungen an die Forschung.

Diese Anforderungen umfassen u. a. die Einhaltung der GWP, das Erzielen belastbarer, reproduzierbarer Ergebnisse sowie eine professionelle Außenwirkung. Dazu zählen Objektivität, die Offenlegung von Interessenkonflikten, die Korrektur von Veröffentlichungen, das Einhalten von Rechtsvorschriften und ein verantwortungsvoller Umgang mit Ressourcen. Qualitativ hochwertige Forschung wird nicht als solche anerkannt, wenn sie mit Ressourcenverschwendung, unzureichenden Stichproben oder unethischen Methoden verbunden ist. Die Erfüllung dieser Anforderungen setzt eine Verpflichtung zur GWP und zum Berufsethos voraus.

Qualitative Ergebnisse entstehen durch methodische Güte, gewartete und kalibrierte Geräte sowie eine sorgfältige Dokumentation und Archivierung. Eine transparente Methodik, Fehlerkorrekturen nach der Veröffentlichung und eine professionelle Rückmeldung tragen ebenfalls zur positiven Außenwirkung bei.

Klar strukturierte und anwenderorientierte Prozesse, zweckorientierte Dokumentation, Selbstüberprüfung und Offenheit, aus Fehlern zu lernen, fördern ein effizientes Ressourcenmanagement. So lassen sich Doppelarbeiten und unnötige Kosten durch fehlerhaftes Material, ungeschultes Personal oder ineffiziente Abläufe verringern.

## Die Grenzen der GWP

Die Einhaltung der GWP ist entscheidend für eine wissenschaftliche Karriere. Sie allein genügt jedoch nicht, um Forschungsqualität konsistent sicherzustellen oder gar zu verbessern. Sie enthält weder konkrete Anforderungen an Prozesse, noch an deren Evaluation oder an Ressourcen – zentrale Aspekte für einen verantwortungsvollen Umgang mit Fördermitteln und für reproduzierbare Ergebnisse. Ihr Verweis auf das Arbeiten *lege artis* bleibt vage und interpretationsabhängig.

**Abb. 2:**
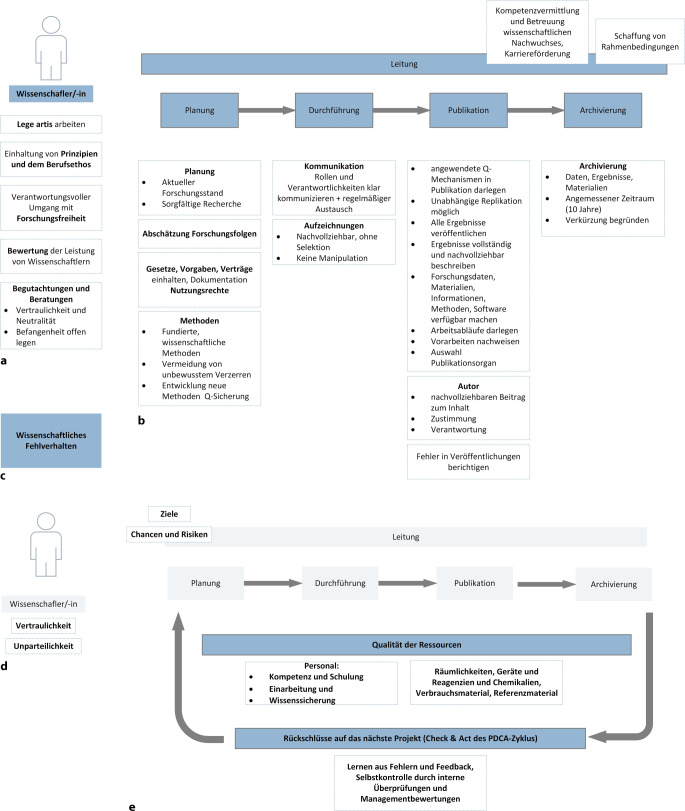
Gute wissenschaftliche Praxis – Anforderungen sortiert und fehlende qualitätsrelevante Kriterien. **a–c** Inhalte der „guten wissenschaftlichen Praxis“ (*GWP*); **a** Anforderungen, die sich direkt an die Wissenschaftlerin/den Wissenschaftler als Person wenden, **b** Leitungs- und Kernprozess eines jeden Forschungslabors bestehend aus Planung, Durchführung, Publikation und Archivierung, **c** Leitlinien für wissenschaftliches Fehlverhalten; **d** und **e** fehlende qualitätsrelevante Kriterien in der GWP, **d** Anforderungen, die sich direkt an die Wissenschaftlerin/den Wissenschaftler als Person wenden, hier fehlen explizit die Vertraulichkeit und Unparteilichkeit der Wissenschaftlerin/des Wissenschaftlers, **e** Leitungs- und Kernprozess eines jeden Forschungslabors, der Rückkopplungsmechanismus aus *Check* und *Act* des Plan-Do-Check-Act-(PDCA-)Zyklus wird nicht näher betrachtet in der GWP; *Q-* Qualitäts-

Abb. 2a–c zeigen die komprimierten DFG-Forderungen, nach Prozessabschnitten sortiert. Diese konzentrieren sich primär auf eine korrekte wissenschaftliche Arbeitsweise und individuelles Verhalten. Abb. 2d, e hingegen verdeutlichen, dass zentrale qualitätsrelevante Elemente von der GWP nicht abgedeckt werden. Es fehlt ein Rückkopplungsmechanismus gemäß dem Plan-Do-Check-Act-(PDCA-)Zyklus, der Qualität aktiv steuerbar macht und kontinuierliche Verbesserung im Forschungsalltag ermöglicht.

Ein solcher Verbesserungsprozess umfasst z. B. das Lernen aus Fehlern, Feedback, Projektreflexion und die regelmäßige kritische Analyse von Arbeitsweisen des Forschungsteams. Gerade im Kontext von Ressourcenverschwendung ist dies essenziell. Zur Sicherstellung qualitativer Forschung sind definierte Laborprozesse unerlässlich, zu denen auch Unterstützungsprozesse gehören, wie z. B. die Einarbeitung neuer Mitarbeitender. Klare Ziele schaffen einen roten Faden und dienen der Priorisierung von Aufgaben. Das Erkennen von Risiken und Chancen unterstützt nicht nur die Zielerreichung, sondern auch die Risikominimierung und die Vermeidung von Ressourcenverschwendung. Auch Anforderungen wie Vertraulichkeit und Unparteilichkeit gehören zur Qualitätsdimension, werden von der GWP jedoch nicht explizit adressiert.

Qualitative Forschungsergebnisse hängen von vielen Faktoren ab, die sich mit den 6M des Ishikawa [14] übersichtlich darstellen lassen:

- *M*ensch: „Organisationen werden nie besser sein als ihre Mitarbeiter“ [15], Kompetenzen, Verantwortlichkeiten, Schulungen,
- *M*ilieu: geeignete Räume, Umweltbedingungen, Lagermöglichkeiten, Trennung von Laborbereichen,
- *M*aterial: Qualität, Lagerung und Handhabung von Proben, Probenahme und Reagenzien,
- *M*ethode: Qualität der Methoden (z. B.: Verifizierung und Validierung),
- *M*aschine: geeignete Geräte, die korrekt messen (z. B.: Kalibrierung, Wartung, Instandhaltung),
- *M*anagement: Einhaltung der GWP, Reflexion der eigenen Forschung und Lernbereitschaft, Anpassungsbereitschaft bei Feedback und Fehlern, definierte Prozesse, klare Verantwortlichkeit, gute Kommunikation.

Die Qualität der eigenen Forschung sicherzustellen, ist mehr als der Verweis auf das Berufsethos. Ein QMS kann hier unterstützen, indem es die unterschiedlichen Prozesse eines Forschungslabors erfasst und diese so wenig wie möglich, aber so viel und Mehrwert bringend wie nötig standardisiert.

## Ein Forschungs-QMS

Ein QMS für Forschungslabore muss flexibel und anpassbar sein – ohne die Starrheit klassischer industrieller Systeme, auch wenn dort zunehmend prozessorientierte Ansätze anstelle einer Produktfokussierung zur Qualitäts- und Effizienzverbesserung eingesetzt werden. Prozessmanagement dient der Steuerung und Optimierung von Abläufen und verbessert die bereichsübergreifende Zusammenarbeit durch eine organisationsweite Betrachtung der Prozesse. Dies ist besonders relevant in komplexen Einrichtungen, wie z. B. Krankenhäusern [16], der öffentlichen Verwaltung [17], Pharmafirmen [18] oder der Luftfahrttechnik [19]. Die Qualität der Produkte oder Dienstleistungen der Einrichtungen wird durch eine Standardisierung und Überwachung der Prozesse erzielt [20]. Im QM steht die Lenkung und Verbesserung der Produkt- oder Dienstleistungsqualität durch eine Betrachtung des gänzlichen Lebenszyklus des Produktes oder der Dienstleistung im Mittelpunkt. Auch hier bildet der Prozessgedanke eine Basis, indem die Tätigkeiten im Lebenszyklus als zusammenhängende, messbare und optimierbare Prozesse verstanden werden, die beständige und vorhersehbare Ergebnisse erzielen [13]. Für das Forschungs-QMS wurden passend zur zumeist geschlossenen Struktur von Forschungsgruppen gezielt Theorien des QM angewendet.

Das RKI hat ein QMS für die Forschung entworfen und gemeinsam mit der Technischen Universität Berlin und der Fraunhofer-Einrichtung für Individualisierte und Zellbasierte Medizintechnik validiert und in Pilotlaboren etabliert. Um sowohl alle qualitätsrelevanten Laborprozesse als auch Forschungscharakteristika zu erfassen und qualitative Anforderungen an diese zu definieren, wurde folgende Literatur berücksichtigt:

- GWP [21],
- DIN EN ISO 15189 – Medizinische Laboratorien – Anforderungen an die Qualität und Kompetenz [22],
- DIN EN ISO/IEC 17025 – Allgemeine Anforderungen an die Kompetenz von Prüf- und Kalibrierlaboratorien [23],
- DIN EN ISO 9001 – Qualitätsmanagementsysteme – Anforderungen [24],
- DFG-Empfehlungen zur Replizierbarkeit [25].

**Abb. 3:**
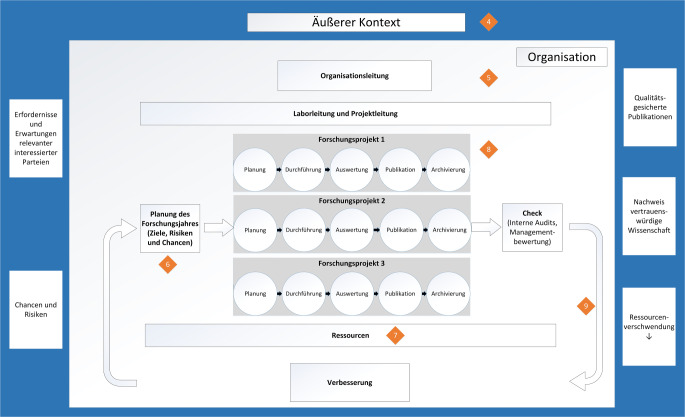
Prozesslandschaft eines Forschungslabors. Orangefarbene Rauten sind die Kapitel des erstellten Anforderungskatalogs für die Forschung: (*4*) allgemeine Anforderungen, (*5*) strukturelle Anforderungen, (*6*) Planung der Forschungsprozesse, (*7*) Anforderungen an Ressourcen, (*8*) Forschungsprojekt, (*9*) Anforderungen an das Managementsystem

Ein Vergleich der Normen ergab einen gemeinsamen Kern von Qualitätsanforderungen, der um forschungsspezifische Aspekte der GWP und DFG-Empfehlungen zur Replizierbarkeit erweitert wurde. Um den in QMS verankerten Auditprozess zu etablieren, diente das Curriculum Ärztliches Peer Review als Orientierung [26]. Die definierten Qualitätsanforderungen für Forschungslabore erfassen qualitätsrelevante Prozesse eines Forschungslabors, einschließlich der Führungs- und Unterstützungsprozesse (Abb. 3 und Onlinematerial 1).

Durch die Einhaltung der Standards wird eine systematische Umsetzung der GWP sowie der bereits genannten „Kunden“-Anforderungen sichergestellt. Die Struktur der Anforderungen orientiert sich an der *High-Level Structure *([27]; Tab. 1).

**Tab. 1:** Selbstauferlegte Forschungsanforderungen

Kapitel	Überschrift	Inhalt
1	Anwendungsbereich	–
2	Normative Verweisungen	–
3	Begriffe	–
4	Allgemeine Anforderungen	Unparteilichkeit, Vertraulichkeit, Bewusstsein der gesetzlichen Rahmenbedingungen und der „guten wissenschaftlichen Praxis“ (GWP), ethisches Verhalten, *Dual Use*
5	Strukturelle Anforderungen	Rechtsträger, Organisationsstrukturen, Anforderungen an die Leitung der Organisation und an die Leitung des Labors, Qualitätsmanagement
6	Planung der Forschungsprozesse	Bestimmung von Zielen für das Forschungslabor, Bestimmung von Risiken und Chancen für die jeweiligen Projekte und Ergreifen von Maßnahmen
7	Anforderungen an Ressourcen	Personal (Schulung, Einarbeitung, Wissenssicherung), Kommunikation, Räumlichkeiten und Umgebungsbedingungen, Ausrüstung, Kalibrierung, metrologische Rückführbarkeit, Reagenzien und Verbrauchsmaterial, extern bereitgestellte Produkte und Dienstleistungen, IT
8	Forschungsprojekt	Planung (Versuchsdesign, Methoden, Verifizierung/Validierung, Messunsicherheit), Durchführung (Dokumentation Untersuchungsverfahren und Aufzeichnungen, Probenahme, Qualitätssicherungsmaßnamen), Auswertung, Publikation (Beschreibung von Methoden, Analysen, Qualitätssicherung, Replizierbarkeit, Zustimmung Autoren, Nachweis Vorarbeiten, Publikationsorgan, Berichtigung veröffentlichter Ergebnisse), Archivierung
9	Anforderungen an das Managementsystem	Lenkung von Dokumenten und Aufzeichnungen, Fehler- und Feedbackmanagement, interne Peer Reviews, Managementreview

Der Standardkatalog unterscheidet sich von den bisher etablierten Normen für Laboratorien durch die spezifischen Anforderungen für die Forschung und die direkte Integration von Forderungen der GWP. Er ist kürzer und enthält weniger strikte Anforderungen an dokumentierte Prozesse. Ziel dieses Dokuments ist es, Forschungslabore anzuregen, ihre Prozesse kritisch zu hinterfragen und zu optimieren, um Ressourcen effizienter einzusetzen. QM kann dabei als Chance verstanden werden, dem Ressourcenmangel wirksam zu begegnen. Bei der Umsetzung der Standards obliegt es den Forschungslaboren selbst, die Anwendbarkeit und Skalierbarkeit der Anforderungen einzuschätzen, um den größtmöglichen Nutzen zu erzielen. Des Weiteren weicht der Standardkatalog von klassischen Dokumentationsvorgaben, wie z. B. von Verfahrensanweisungen für die Auswahl von Dienstleistern, ab. Im Folgenden werden ausgewählte Elemente des QMS und deren Umsetzung erläutert:

- *Vorgabedokumente* (Standard Operating Procedures (SOPs), Arbeitsanweisungen etc.) sind auch im Forschungskontext wertvoll. Sie unterstützen Mitarbeitende, die Methoden oder Prozesse (z. B. Beschaffung) nicht regelmäßig durchführen, etwa bei Krankheits- oder Urlaubsvertretungen, sichern die Nachvollziehbarkeit von Arbeitsschritten über längere Zeiträume, erleichtern die Einarbeitung neuer Teammitglieder und sichern Wissen, besonders bei häufigem Personalwechsel. Um Kreativität und zufällige Entdeckungen nicht zu behindern, sollten Vorgabedokumente den allgemeinen Ablauf einer Methode knapp und praxisnah beschreiben. Zu detaillierte oder unnötige Anweisungen bleiben oft ungenutzt. Wesentlich ist die klare Kommunikation über Existenz, Inhalt und Ablageort der Dokumente sowie die Sicherstellung ihrer Aktualität. Veraltete Versionen sind zu archivieren, nur aktuelle dürfen verwendet werden. Die Erstellung sollte mindestens im 4‑Augen-Prinzip erfolgen: Eine zweite Person überprüft Inhalt und Verständlichkeit. Änderungen sollten markiert werden, damit die Anpassungen auf den ersten Blick erkennbar sind. Besonders geeignet für eine strukturierte Lenkung sind Prozessbeschreibungen organisatorischer Abläufe (z. B. *Onboarding*), Anleitungen zur Gerätebedienung sowie Standardmethoden (z. B. Western Blots). Bei Methoden ohne eigene Arbeitsanweisung erfolgt die Dokumentation im Laborbuch – detailliert und nachvollziehbar. Klare Vorgaben der Forschungsleitung können helfen, relevante Informationen in einer Weise zu dokumentieren, die von anderen Teammitgliedern verstanden und genutzt werden kann. Ein elektronisches Laborbuch erleichtert dies.
- *Der sachgerechte Umgang mit Reagenzien und Verbrauchsmaterial* und die Sicherstellung der Qualität dieser Materialien sind auch in Forschungslaboren essenziell, um verlässliche Ergebnisse zu erzielen. Fehlerhafte Lagerung kann zu Qualitätsverlust oder Unbrauchbarkeit führen. Ein überschrittenes Haltbarkeitsdatum bedeutet jedoch nicht automatisch, dass Materialien entsorgt werden müssen. Im Rahmen eines QMS kann ihre weitere Nutzung nach risikobasierter Bewertung entschieden werden. Es sollte auch abgewogen werden, ob nicht eine Testung zur Brauchbarkeit des Reagenzes vor der Anwendung an der eigentlichen Probe sinnvoll sein kann. Eine kurze Dokumentation, die festhält, wann das Reagenz zuletzt erfolgreich verwendet wurde, kann für alle Mitarbeitenden eine wertvolle Orientierung bieten.
- *Das Lernen aus Fehlern* ist ein wichtiger Schlüssel zu effizienteren Prozessen. Daher sollte eine offene Fehlerkultur etabliert werden, die den Fokus nicht auf die Schuldfrage legt, sondern auf die Ursachenanalyse. Ein strukturiertes Fehlermanagement kann dabei hilfreich sein. Dieses umfasst eine zentrale und prägnante Dokumentation folgender Punkte:
  - Was ist passiert?
  - Welche Sofortmaßnahmen wurden ergriffen?
  - Welche Konsequenzen hatte das Ereignis für die Forschung (z. B. müssen Experimente wiederholt werden)?
  - Warum konnte der Fehler auftreten?
  - Wie wurde die Ursache behoben und war die Maßnahme erfolgreich?
  Durch diese Dokumentation können Prozesse identifiziert werden, die wiederholt zu Problemen führen, und bei erneut auftretenden Fehlern lassen sich frühere Lösungswege nachverfolgen – besonders hilfreich bei häufig wechselndem Personal. Dabei sollte beachtet werden, dass Forschung naturgemäß Unregelmäßigkeiten aufweist. Dokumentiert werden sollten nur Vorfälle, die den Forschungsablauf spürbar beeinträchtigen, wie z. B. Kühlschrankausfälle, Wiederholung von Experimenten wegen unzureichender Dokumentation oder vergessener Standards sowie die Unbrauchbarkeit von Reagenzien durch falsche Lagerung. Eine einfache Excel-Tabelle kann ein effektives Fehlermanagement ermöglichen, ohne den Aufwand und die Komplexität umfangreicher Unterschriftenwege oder Formulare.
- *Bei internen Peer Reviews* in Forschungslaboren diente das erprobte Curriculum Ärztliches Peer Review [26] als Orientierung. So sollten die Reviews analog auf Freiwilligkeit und Sanktionsfreiheit beruhen. Sie unterschieden sich deutlich von Audits in medizinischen Laboren oder der Industrie. Statt strenger Normprüfung stand der kollegiale Austausch auf Augenhöhe im Fokus. Der bereichsübergreifende Austausch, etwa zur Forschungsorganisation, konnte wertvolle Impulse liefern und neue Perspektiven eröffnen. Fehlerhafte, redundante oder unnötig komplexe Prozesse wurden erkannt und korrigiert. Anstelle eines starren Auditberichts mit verpflichtenden Umsetzungsfristen sollten Empfehlungen, z. B. als GAP-Analyse (Soll-Ist-Vergleich), ausgesprochen werden. Am RKI erfolgte die Bewertung der festgestellten Lücken mit einem Ampelsystem, ergänzt durch konkrete Handlungsempfehlungen. Die Ergebnisse wurden gemeinsam mit den Forschungsteams ausgewertet. Bereits durch die Gespräche identifizierten die Labore eigenständig Punkte, die sie umsetzen wollten. Die Reviews schärften das Bewusstsein für den gesamten Forschungsprozess und deckten Schwachstellen auf, die im Alltag oft übersehen wurden.

## Interne Peer Reviews in Forschungsteams

Um die Anwendbarkeit der definierten Anforderungen zu überprüfen und den Istzustand der Forschungslabore im Hinblick auf diese Anforderungen zu erheben, wurden 5 biomedizinische Forschungsteams unterschiedlicher Organisationen und Mitarbeiteranzahl untersucht. Ausgangspunkt war eine vom Laborleitenden ausgewählte Publikation des Forschungsteams, zu der dieser auch auskunftsfähig war.

Zur Vergleichbarkeit der Labore wurden aus dem Standardkatalog spezifische Fragen abgeleitet und anhand von Beispielen aus der Publikation konkretisiert, etwa ob ein erwähntes Standardprotokoll noch verfügbar ist („Genom- und Plasmid-DNA wurden gemäß Standardprotokollen hergestellt, amplifiziert und sequenziert“) oder ob aus Grafiken auf die zugrunde liegenden Rohdaten geschlossen werden kann.

Ergänzend wurden übergreifende Fragen gestellt, z. B.:

- Ist das Verfahren bei Verdacht auf wissenschaftliches Fehlverhalten bekannt?
- Wie erfolgen Einarbeitung und Wissenssicherung?
- Wie wird mit erhobenen Daten umgegangen?
- Wie wird mit Fehlern und Feedback verfahren?

**Abb. 4:**
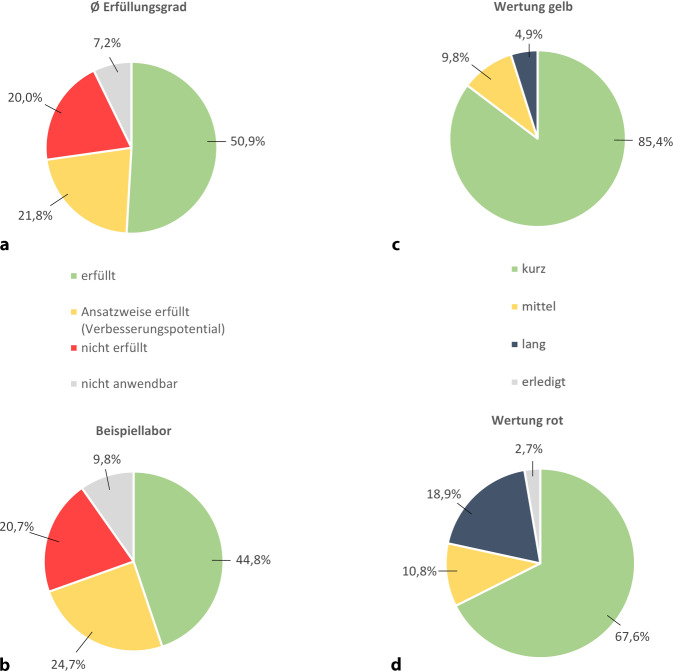
**a** Durchschnittlicher Erfüllungsgrad der QM-Anforderungen, *n* = 5 Labore aus 2 unterschiedlichen Forschungsorganisationen; **b** Ergebnis des internen Peer Review des Labors; **c,** **d** Selbsteinschätzung der Umsetzbarkeit der QM-Anforderungen eines Forschungslabors, **c** Wertung der ansatzweise erfüllten Forderungen, **d** Wertung der nicht erfüllten Forderungen

Im Schnitt erfüllten die Labore 50,9 % der Anforderungen (Abb. 4a). Häufig erfüllt wurden z. B.:

- vertraulicher Umgang mit persönlichen Daten,
- klar definierte Zuständigkeiten der Laborleitenden,
- Eignung, Angemessenheit und Instandhaltung der Räumlichkeiten,
- ehrliche und vollständige Dokumentation,
- angemessene Archivierungsdauer von Dokumenten,
- Einhaltung der GWP im Publikationsprozess.

Hingegen traten bei folgenden Punkten häufig Mängel auf:

- Autorisierungen und Verantwortlichkeiten,
- Einarbeitung und Wissenssicherung,
- Vorgaben für Aufzeichnungen und Ablage,
- Dokumentenmanagement,
- Überwachungen von Umgebungsbedingungen,
- Kalibrierung und Wartung,
- Fehler- und Feedbackmanagement.

Auch bei den Punkten Unparteilichkeit und *Dual Use* sahen die Laborleitenden entweder keine Relevanz für ihre Arbeitsgruppe oder ordneten die Verantwortung der übergeordneten Organisation zu.

Als nicht anwendbare Anforderungen des Standards wurde von den befragten Laboren z. B. die Primärprobenahme identifiziert, da diese nicht selbst erfolgt, sondern von externen Kliniken übernommen wird, die über eigene Qualitätssicherungssysteme verfügen. Dennoch stellt die Präanalytik eine kritische Schwachstelle im Forschungsprozess dar [28]. Daher sollten Forschungslabore bei der Wahl von Kooperationspartnern auf deren Qualitätssicherung und auf die Beschaffenheit der Probe (z. B. ordnungsgemäße Beschriftung, korrektes Gefäß, Sichtprüfung) achten. Minderwertige Proben können zu fehlerhaften Ergebnissen und unnötigem Ressourcenverbrauch führen. Wo möglich, sollte die Qualität der Probenahme aktiv abgesichert werden.

Nach der Erstellung der GAP-Analyse wurde diese mit den jeweiligen Laborleitenden besprochen. Dabei wählten die Labore unterschiedliche Ansätze, um die vorgeschlagenen Verbesserungsmaßnahmen umzusetzen. Ein Labor (Abb. 4b) bewertete z. B. die Maßnahmen eigenständig und schätzte die voraussichtliche Umsetzungsdauer ein, um Prioritäten zu setzen. Die Ergebnisse dieses Vorgehens (Abb. 4c, d) zeigen, dass weit über die Hälfte der vorgeschlagenen Maßnahmen für dieses Labor kurzfristig realisierbar sind.

## Praktiken von Forschungslaboren

Die Gespräche mit den Forschungsteams und die Analyse ihrer Prozesse zeigten häufig ähnliche Praktiken und Herausforderungen auf. Einige davon sind in Tab. 2 dargestellt, einschließlich typischer Probleme und möglicher Verbesserungsvorschläge.

**Tab. 2:** Angewohnheiten von Forschungslaboren

*Erfahrung*	*Unübersichtliche Ablage von Aufzeichnungen und Vorgabedokumenten*
Beispiele	Standard Operating Procedures (SOPs) über mehrere Speicherorte verteilt zwischen anderen Dokumenten; alle in einem Ordner, aber keine eindeutige Benennung; manche als Textdatei, andere als Präsentation
Problem	Zu hoher Zeitaufwand, um das Dokument herauszusuchen, uneinheitliche Dateiformate ohne gemeinsames Layout kosten dadurch Zeit, dass sich das Personal erst in ihnen zurechtfinden muss, Arbeitsanweisungen werden missachtet
Verbesserung	Geordnete und intuitive Ablage; eindeutige, kurze Benennung des Dokuments; einheitliche Formatvorlagen für ein schnelles Zurechtfinden des Personals
*Erfahrung*	*Fehlende Schulung der Dokumente*
Beispiele	Mitarbeitenden ist nicht bekannt, für welche Methoden und Prozesse es Anweisungen gibt
Problem	Arbeitsanweisungen werden aufgrund von Unwissenheit missachtet
Verbesserung	Schulung von Dokumentenänderungen im Labormeeting; Aufnahme der Dokumente in den Einarbeitungsprozess neuer Mitarbeitender
*Erfahrung*	*Falsche Einschätzung des Teams*
Beispiele	Annahme, dass das Team keine Änderungen im Ablauf haben möchte oder QM im Team nicht gewollt sei
Problem	Keine Verbesserungen
Verbesserung	Offene Kommunikation über mögliche Verbesserungsmaßnahmen
*Erfahrung*	*Mangelndes Bewusstsein bei Qualitätsaspekten*
Beispiele	Keine Temperaturüberwachung in Kühlschränken, Brutschränken, Räumen; Verlass auf Technik des Herstellers
Problem	Gerätefehler fällt zu spät auf, interne Temperaturmesssysteme können auch fehlerhaft sein, im schlimmsten Fall Proben/Materialien zerstört; Proben/Materialien etc. auf Verwendbarkeit zu testen ist zeitintensiv; Raumtemperatur kann stark variieren und bei temperaturempfindlichen Verfahren beeinflussend wirken
Verbesserung	Keine Einsparung bei Qualität, Anschaffung von Überwachungssystemen, z. B. Temperaturlogger
*Erfahrung*	*Besprechungen in zu großen Teams*
Beispiele	Teilnahme von Fachgebietsleiter, Laborleiter und anderen Arbeitsgruppen
Problem	Zu hohe Hemmschwelle, Probleme und missglückte Experimente anzusprechen
Verbesserung	Überlegung, wer in dem Meeting wirklich teilnehmen muss; für Verbesserungsmeetings kleine Kreise ansetzen
*Erfahrung*	*Fehlende Vorgaben im Team für Aufzeichnungen*
Beispiele	Aufzeichnungen werden von den einzelnen Mitarbeitenden unterschiedlich gemacht; individuelles Ablagesystem wird geschaffen
Problem	Einlesen in die Methoden ist für neue Mitarbeitende schwierig; Daten‑/Informationsverlust
Verbesserung	Vorgaben zu Dokumentation im Team besprechen; Dokumentationsformblatt entwerfen, um Datenverlust entgegenzuwirken
*Erfahrung*	*Chargenwechsel werden nicht dokumentiert*
Beispiele	Es wird nur der Name der Firma angegeben, mit der anfangs gearbeitet wurde
Problem	Datenverlust und Fehlersuche erschwert
Verbesserung	Dokumentationsformblatt oder Checkliste für richtiges Dokumentieren
*Erfahrung*	*Vorstellung von Ergebnissen in Teambesprechungen als Präsentation*
Beispiele	Präsentation über getätigte Forschungsarbeit erstellt und vorgetragen
Problem	Es werden nur die Erfolge gezeigt und nicht das, was misslungen ist; kein Wissens- und Erfahrungsaustausch; keine Fehleranalyse möglich
Verbesserung	Arbeitsbesprechungen anhand von Laborbuch und nicht anhand einer extra gefertigten Präsentation
*Erfahrung*	*Fehlende Kalibrierung an qualitätsrelevanten Geräten*
Beispiele	Brutschränke werden nicht regelmäßig kalibriert (CO_2_, Temperatur)
Problem	Experimente schlagen fehl; Materialverschwendung; Zeitverschwendung
Verbesserung	Kalibrierungen an qualitätsrelevanten Geräten einführen

Es wurde deutlich, dass selbst innerhalb einer Organisation Abläufe stark variieren. Viele Forschungsteams haben im Laufe der Zeit basierend auf individuellen Erfahrungen und Lektionen eigene Strategien und Prozesse entwickelt.

Allerdings zeigte sich, dass es zwischen den Forschungsleitenden kaum einen Austausch darüber gibt, wie Prozesse sinnvoll gestaltet oder Labore effizient gemanagt werden können. Dieses Kommunikationsdefizit führt dazu, dass Labore ähnliche Herausforderungen isoliert bewältigen und jeweils eigene Lösungswege entwickeln. Ein gemeinsamer Austausch über Fehler, Reaktionen und ergriffene Maßnahmen könnte jedoch für andere Teams wertvoll sein – unabhängig vom Forschungsschwerpunkt. Ein solches Format ließe sich analog zu QM-Strukturen von akkreditierten Laboren etwa in Form von Qualitätszirkeln organisieren.

Die befragten Laborleitenden zeigten sich grundsätzlich offen für einen solchen Austausch und erkannten dessen potenziellen Nutzen an. Sie äußerten Neugier darüber, wie andere Forschungslabore ihre Prozesse steuern und Hindernisse bewältigen. Zugleich wurde jedoch betont, dass Forschungslabore oft wenig transparent agieren. Die Leitenden äußerten Zweifel an der Umsetzbarkeit eines offenen Austauschs, da viele Labore zögern, ihre internen Vorgehensweisen und Schwachstellen offenzulegen. Transparenz werde von Forschungslaboren oft als Risiko wahrgenommen.

Obwohl der Wunsch nach einer Kommunikation über Fehler und Lösungen groß ist, gilt diese in der Praxis aktuell noch als schwer umsetzbar. Für etwaige Qualitätszirkel betonten die Laborleitungen, dass ein ausgewogenes Kosten-Nutzen-Verhältnis wichtig sei. Ein Vorschlag war, solche Zirkel zu nutzen, um die GAP-Analyse des internen Peer Review abzugleichen und gemeinsam Strategien zur Verbesserung zu entwickeln.

Die Forschungsleitungen zeigten sich gegenüber internen Peer Reviews aufgeschlossen. Da Peer Reviews bei der Bewertung wissenschaftlicher Publikationen fest etabliert sind, wurde ihre Ausweitung auf Forschungsprozesse als gewinnbringende Maßnahme wahrgenommen. Dank Freiwilligkeit und Sanktionsfreiheit ergaben sich für die Labore ausschließlich Vorteile, etwa neue Impulse und praxisnahe Verbesserungen. Bereits das ärztliche Peer Review, das zu Beginn des 20. Jahrhunderts in den USA entstand [26], zeigte schon vor Jahrzehnten, dass strukturierter, praxisrelevanter, kollegialer Austausch und Feedback zu wirksamen Änderungen und Qualitätsverbesserungen führen können [29, 30]. Heute ist es ein etabliertes Instrument der Qualitätssicherung in Kliniken [31, 32] und im ambulanten Bereich [33]. Zentrale Elemente wie Selbst- und Fremdreflexion sind auch auf Forschungslabore übertragbar – durch kollegiale Begutachtung sowie strukturierte Selbsteinschätzung mithilfe von Evaluierungsbögen.

## Selbstbewertung der eigenen Prozesse

Auffällig war, dass die Forschungsteams für gleiche Prozesse teils unterschiedliche Vorgehensweisen anwenden. Diese sind teils besser, teils schlechter geeignet und oft historisch gewachsen. Zur Bewertung der Qualität und Reife dieser Prozesse wurde ein Selbstbewertungstool entwickelt (s. Onlinematerial 2). Es ermöglicht Forschenden, eigenständig den Reifegrad ihrer Forschungsprozesse zu evaluieren, Verbesserungspotenziale zu erkennen, Prioritäten zu setzen und Maßnahmen abzuleiten.

Das Tool basiert auf ausgewählten Aspekten der Qualitätsanforderungen und den unterschiedlichen Vorgehensweisen der Forschungsteams, die bei der Entwicklung der Abstufung berücksichtigt wurden. Es liefert eine Übersicht über Stärken und Schwächen sowie den Reifegrad der Forschungsprozesse. Bei wiederholter Anwendung lässt sich zudem der Fortschritt über die Zeit nachvollziehen. Das Selbstbewertungstool orientiert sich an DIN EN ISO 9004, Anhang A – Werkzeug zur Selbstbewertung [34].

## Ausblick und Fazit

So vielversprechend die Theorie eines QMS auch ist, ein solches System muss gelebt werden. Wird es dem Team lediglich aufgezwungen und bleibt der Nutzen für die Mitarbeitenden unklar, kann es schnell zur Belastung werden. Besonders in der Forschung gilt es, unnötige Belastungen durch Bürokratie und Mehraufwand zu vermeiden. Erkennt das Labor dagegen aber das QM als Chance an, wird es einen ressourcenoptimierten Einsatz erzielen.

In Pilotlaboren führt das RKI eine anonyme Vorher-Nachher-Mitarbeiterbefragung durch, in der alle Teammitglieder zu ihren Einstellungen und Erfahrungen rund um Qualität und QM befragt werden. Ziel ist es, herauszufinden, wie gut die Mitarbeitenden mit den Prozessen in ihrem Forschungslabor zurechtkommen, wo Verbesserungspotenziale bestehen und wie das QM insgesamt wahrgenommen wird.

Während ein QMS in medizinischen Laboren gesetzlich verpflichtend ist, stellt es in Forschungslaboren oft Neuland dar. In der Forschung sollten diese Systeme vor allem Arbeitsabläufe optimieren und die Qualität verbessern. Qualitativ hochwertige Forschung hängt nicht nur vom Einhalten beruflicher Kodizes und Gesetze ab, sondern auch von weiteren Faktoren wie Personal, Geräten, Reagenzien, Verbrauchsmaterialien und anderen Ressourcen. Managementmethoden wie Zieldefinitionen, Risiko- und Chancenbewertung, Feedback- und Fehlermanagement sowie die Festlegung von klaren Prozessen und regelmäßige Überprüfungen tragen zur Effizienzsteigerung des Labors bei.

Das vorgestellte QMS soll helfen, diese einzelnen Prozesse in Forschungslaboren zu erfassen und ein gemeinsames Qualitätsverständnis zu fördern. Mit dem Selbstbewertungstool können Forschungsteams ihre Prozessqualität bewerten und Anregungen für Verbesserungen erhalten.

## Supplementary Information

Onlinematerial 1

Onlinematerial 2

## References

[1] Fleming A (1945) Penicillin. In: Nobel Lectures – Physiology or Medicine 1942–1962. Elsevier, Amsterdam

[2] Anonymous (2020) 125 Jahre Röntgenstrahlung: Eine zufällige Sternstunde der Medizin. https://www.aerzteblatt.de/nachrichten/117916/125-Jahre-Roentgenstrahlung-Eine-zufaellige-Sternstunde-der-Medizin. Zugegriffen: 10. Jan. 2025

[3] Dirnagl U, Kurreck C, Castaños-Vélez E, Bernard R (2018) Quality management for academic laboratories: burden or boon? EMBO Rep 19:e47143. 10.15252/embr.20184714310.15252/embr.201847143 30341068 10.15252/embr.201847143 PMC6216282

[4] Martínez-Perales S, Ortiz-Marcos I, Ruiz JJ (2021) A proposal of model for a quality management system in research testing laboratories. Accredit Qual Assur 26:237–248. 10.1007/s00769-021-01479-3

[5] Hewera M, Nickel AC, Knipprath N et al (2020) An inexpensive and easy-to-implement approach to a quality management system for an academic research lab. F1000Res 9:660. 10.12688/f1000research.24494.2 32765843 10.12688/f1000research.24494.1 PMC7385541

[6] Weinert S, Wohlfahrt I, Schmid A, Frohme M (2010) Einführung eines Qualitätsmanagement-Systems in einem molekularbiologischen Labor einer Hochschule. Wiss Beitr 14:49–56. 10.15771/0949-8214_2010_1_6

[7] Brünschwitz S, Kleymann-Hilmes J (2024) Vorteile und Ansätze eines Qualitätsmanagementsystems in biomedizinischen Forschungslaboratorien. Bundesgesundheitsblatt Gesundheitsforschung Gesundheitsschutz 67:99–106. 10.1007/s00103-023-03797-y 37982816 10.1007/s00103-023-03797-y PMC10776475

[8] Deutsche Forschungsgemeinschaft (2022) Gute wissenschaftliche Praxis. https://www.dfg.de/foerderung/grundlagen_rahmenbedingungen/gwp/index.html. Zugegriffen: 25. Nov. 2022

[9] ALLEA (2023) The European code of conduct for research integrity—revised edition 2023. https://allea.org/portfolio-item/european-code-of-conduct-2023/. Zugegriffen: 10. Jan. 2025

[10] Deutsche Forschungsgemeinschaft (2019) Kodex – Leitlinien zur Sicherung guter wissenschaftlicher Praxis. https://wissenschaftliche-integritaet.de/. Zugegriffen: 24. Febr. 2025

[11] Van Noorden R (2023) More than 10,000 research papers were retracted in 2023—a new record. Nature 624:479–481. 10.1038/d41586-023-03974-8 38087103 10.1038/d41586-023-03974-8

[12] Baker M (2016) 1,500 scientists lift the lid on reproducibility. Nature 533:452–454. 10.1038/533452a 27225100 10.1038/533452a

[13] Deutsches Institut für Normung e. V. (2015) DIN EN ISO 9000:2015-11 Qualitätsmanagementsysteme – Grundlagen und Begriffe. https://www.dinmedia.de/de/norm/din-en-iso-9000/235671064. Zugegriffen: 9. Apr. 2025

[14] Bundesministerium des Innern und für Heimat (2025) Ursache-Wirkungs-Diagramm. https://www.orghandbuch.de/Webs/OHB/DE/Organisationshandbuch/6_MethodenTechniken/63_Analysetechniken/632_Ursache-Wirkungs-Diagramm/ursache-wirkungs-diagramm-node.html. Zugegriffen: 10. Jan. 2025

[15] Volkmer R (2019) Organisationen werden nie besser sein als ihre Mitarbeiter. https://leanbase.de/publishing/post/organisationen-werden-nie-besser-sein-als-ihre-mit. Zugegriffen: 10. Apr. 2024

[16] Braun S (2017) Prozessmanagement: Wie Kliniken organisatorische Defizite effizient beheben. Dtsch Ärztebl

[17] Bundesministerium des Innern und für Heimat (2025) Prozessmanagement in der öffentlichen Verwaltung. https://www.bmi.bund.de/DE/themen/moderne-verwaltung/verwaltungsmodernisierung/qualitaetsmanagement/prozessmanagement/prozessmanagement-node.html. Zugegriffen: 9. Apr. 2025

[18] Sanofi (2024) Internal control and processes. https://www.sanofi.com/assets/dotcom/content-app/documents/Internal-Control-and-Processes_2024.pdf. Zugegriffen: 15. Apr. 2025

[19] Lufthansa Technik (2025) Quality Management. https://www.lufthansa-technik.com/en/quality. Zugegriffen: 9. Apr. 2025

[20] Funke J (2023) Effektives Prozessmanagement: Optimierte Abläufe für mehr Effizienz, Qualität und Wettbewerbsfähigkeit. https://www.it-p.de/blog/effektives-prozessmanagement/. Zugegriffen: 9. Apr. 2025

[21] Deutsche Forschungsgemeinschaft (2019) Guidelines for safeguarding good research practice – code of conduct. https://zenodo.org/record/3923602. Zugegriffen: 29. Jan. 2022

[22] Deutsches Institut für Normung e. V. (2024) DIN EN ISO 15189:2024-08 Medizinische Laboratorien – Anforderungen an die Qualität und Kompetenz. https://www.dinmedia.de/de/norm/din-en-iso-15189/375920985. Zugegriffen: 9. Apr. 2025

[23] Deutsches Institut für Normung e. V. (2018) DIN EN ISO/IEC 17025:2018-03 Allgemeine Anforderungen an die Kompetenz von Prüf- und Kalibrierlaboratorien. https://www.dinmedia.de/de/norm/din-en-iso-iec-17025/278030106. Zugegriffen: 9. Apr. 2025

[24] Deutsches Institut für Normung e. V. (2015) DIN EN ISO 9001:2015–11 Qualitätsmanagementsysteme – Anforderungen. https://www.dinmedia.de/de/norm/din-en-iso-9001/235671251. Zugegriffen: 9. Apr. 2025

[25] Ständige Senatskommission für Grundsatzfragen in der Klinischen Forschung (2018) Replizierbarkeit von Ergebnissen in der Medizin und Biomedizin; Stellungnahme der Arbeitsgruppe „Qualität in der Klinischen Forschung“ der DFG-Senatskommission für Grundsatzfragen in der Klinischen Forschung. https://www.dfg.de/download/pdf/dfg_im_profil/geschaeftsstelle/publikationen/stellungnahmen_papiere/2018/180507_stellungnahme_replizierbarkeit_sgkf.pdf. Zugegriffen: 25. Nov. 2022

[26] Bundesärztekammer (2013) Curriculum Ärztliches Peer Review. https://www.bundesaerztekammer.de/fileadmin/user_upload/_old-files/downloads/CurrAerztlPeerReview2013.pdf. Zugegriffen: 8. Aug. 2023

[27] TÜV Nord (2021) High-Level-Structure sorgt für Vereinheitlichung. https://www.tuev-nord.de/de/unternehmen/zertifizierung/news/artikel/article/high-level-structure-sorgt-fuer-vereinheitlichung/. Zugegriffen: 9. Apr. 2025

[28] Bonini P, Plebani M, Ceriotti F, Rubboli F (2002) Errors in laboratory medicine. Clin Chem 48:691–698. 10.1093/clinchem/48.5.691 11978595

[29] Grol R (1994) Quality improvement by peer review in primary care: a practical guide. Qual Health Care 3:147–152. 10.1136/qshc.3.3.147 10139412 10.1136/qshc.3.3.147 PMC1055218

[30] Grol R, Mokkink H, Schellevis F (1988) The effects of peer review in general practice. J R Coll Gen Pract 38(306):10–13 3204541 PMC1711379

[31] Helios Kliniken GmbH (2025) Wie kommt Innovation ans Patientenbett? https://www.helios-gesundheit.de/unternehmen/ueber-helios/schwerpunkte/wissenschaft-forschung/dossier-forschung/innovationen-am-patientenbett/. Zugegriffen: 9. Apr. 2025

[32] Blum K (2002) Qualitätsverbesserung durch klinische Audits – Evaluation des BMG-Audit-Projektes. Gesundheitsökon Qualitätsmanag 7(6):373–380. 10.1055/s-2002-36150

[33] Kassenärztliche Bundevereinigung (2017) Peer-Review-Verfahren in der vertragsärztlichen Versorgung – Empfehlungen für Praxen, Arztnetze und Qualitätszirkel. https://www.kbv.de/media/sp/Empfehlungen_zu_Peer-Review-Verfahren_in_der_vertragsaerztlichen_Versorgung.pdf. Zugegriffen: 9. Apr. 2025

[34] Deutsches Institut für Normung e. V. (2018) DIN EN ISO 9004:2018-08 Qualitätsmanagement – Qualität einer Organisation – Anleitung zum Erreichen nachhaltigen Erfolgs. https://www.dinmedia.de/de/norm/din-en-iso-9004/283875061. Zugegriffen: 3. Apr. 2025

