# Supplementary material for: More efficient use of resources in research laboratories through quality management
Source: Bundesgesundheitsblatt Gesundheitsforschung Gesundheitsschutz. 2025 Jun 5;68(11):1337–47. [Article in German] doi: 10.1007/s00103-025-04074-w (PMC12583365; doi:10.1007/s00103-025-04074-w)
Supplement: Supplementary file 1 — Onlinematerial 1 [file 103_2025_4074_MOESM1_ESM.pdf]

Anforderungskatalog - Qualitätsanforderungen für Forschungslabore

Inhaltsverzeichnis

Einleitung 4
1 Anwendungsbereich 4
2 Normative Verweisungen 4
3 Begriffe 4
4 Allgemeine Anforderungen 5
4.1 Unparteilichkeit 5
4.2 Vertraulichkeit 6
4.3 Bestimmung des Kontexts 6
4.4 Verpflichtung zur Guten Wissenschaftlichen Praxis 7
4.5 Ethisches Verhalten 8
5 Strukturelle Anforderungen 9
5.1 Rechtsträger 9
5.2 Laborleiter 9
5.3 Organisationsstrukturen 9
5.4 Anforderungen an die Leitung der Organisation 10
5.5 Anforderungen an die Leitung des Labors 10
5.6 Qualitätsmanagement 11
6 Planung der Forschungsprozesse 13
6.1 Ziele 13
6.2 Maßnahmen zum Umgang mit Risiken und Chancen 13

7 Anforderungen an Ressourcen 14
7.1 Allgemeines 14
7.2 Personal 14
7.3 Kommunikation 17
7.4 Räumlichkeiten und Umgebungsbedingungen 18
7.5 Ausrüstung 20
7.5.1 Allgemein 20
7.5.2 Anforderungen 21
7.6 Kalibrierung der Geräte 23
7.7 Metrologische Rückführbarkeit 25
7.8 Reagenzien und Verbrauchsmaterial 25
7.9 Extern bereitgestellte Produkte und Dienstleistungen 28
7.10 Informationsmanagement des Laboratoriums 29
8 Forschungsprojekt 29
8.1 Planung eines Forschungsprojekts 29
8.1.1 Allgemeines 29
8.1.2 Planung und Versuchsdesign 30
8.1.3 Auswahl der Methoden 31
8.1.4 Verifizierung 32
8.1.5 Validierung 33
8.1.6 Messunsicherheit von gemessenen Größenwerten 33
8.2 Durchführung 34
8.2.1 Allgemeines 34

8.2.2 Dokumentation der Untersuchungsverfahren ... 34
8.2.3 Probenahme ... 35
8.2.4 Aufzeichnungen ... 37
8.2.5 Qualitätssicherungsmaßnahmen ... 38
8.3 Auswertung ... 39
8.4 Publikation ... 39
8.4.1 Beschreibung der Methoden und Analysen ... 39
8.4.2 Beschreibung Qualitätssicherung ... 40
8.4.3 Replizierbarkeit ... 41
8.4.4 Nachweis von Vorarbeiten ... 41
8.4.5 Zustimmung der Autoren ... 41
8.4.6 Publikationsorgan ... 42
8.4.7 Berichtigung veröffentlichter Ergebnisse ... 42
8.5 Archivierung ... 43
9 Anforderungen an das Managementsystem ... 43
9.1 Lenkung von Dokumenten des Managementsystems ... 43
9.2 Lenkung von Aufzeichnungen ... 45
9.3 Nichtkonformitäten und Korrekturmaßnahmen ... 45
9.4 Internes Peer Review ... 47
9.5 Managementbewertung ... 47
9.6 Verbesserung ... 49

Einleitung

Dieses Dokument wurde mit dem Ziel entwickelt, das Vertrauen in die Arbeit von Forschungslaboratorien zu fördern. Es enthält Standards für Forschungslaboratorien, damit diese systematisch ihre Prozesse analysieren, verbessern und weiterentwickeln können. Laboratorien, welche dieses Dokument erfüllen, werden auch allgemein in Übereinstimmung mit den Grundsätzen von ISO/IEC 17025 und ISO 9001 arbeiten.

In roter Schrift geschrieben sind die grundlegenden Anforderungen der Leitlinien zur Sicherung guter wissenschaftlicher Praxis (GWP).

Die in dieser Arbeit verwendeten Personenbezeichnungen beziehen sich auf alle Geschlechter.

1 Anwendungsbereich

Dieser Anforderungskatalog legt Standards an die Qualität und Kompetenz von Forschungslaboratorien fest, die anhand von experimentellen Versuchen an menschlichen, tierischen, mikrobiologischen oder nicht lebensfähigen Proben (Gewässer, Boden, Luft, etc.) neues Wissen generieren oder replizieren.

2 Normative Verweisungen

- Leitlinien zur Sicherung guter wissenschaftlicher Praxis – Kodex, Deutsche Forschungsgemeinschaft, September 2019, DOI: 10.5281/zenodo.14281892; Verweis im Text mit „GWP“ und entsprechende Leitliniennummer
- Replizierbarkeit von Ergebnissen in der Medizin und Biomedizin; Stellungnahme der Arbeitsgruppe „Qualität in der Klinischen Forschung“ der DFG-Senatskommission für Grundsatzfragen in der Klinischen Forschung, Ständige Senatskommission für Grundsatzfragen in der Klinischen Forschung, Deutsche Forschungsgemeinschaft, März 2018; Verweis im Text mit „DFG“

3 Begriffe

- **Abweichung:** Synonym zu Nichtkonformität, Nichterfüllung einer Forderung
- **Autor/-in**: ist, wer einen genuinen, nachvollziehbaren Beitrag zu dem Inhalt einer wissenschaftlichen Text-, Daten- oder Softwarepublikation geleistet hat. Sie besitzen eine gemeinsame Verantwortung für Publikation (es sei denn, es wird explizit anders ausgewiesen). (GWP 14).
- **Dokumentenlenkung**: Verfahren zur Erstellung, Genehmigung und Freigabe, Kennzeichnung, Verteilung und Aktualisierung von Dokumenten
- **Dual Use**: Forschungsergebnisse können sowohl Chancen eröffnen als auch Missbrauch unterliegen, bspw. können Forschungen für die Bekämpfung von Bakterien, Viren und Pilzen missbraucht werden, um die Pathogenität zu steigern und gezielt Ausbrüche zu steuern

- **Oberste Leitung:** Person oder Personengruppe, die eine Organisation auf der obersten Ebene führt und steuert (ISO 9000)
- **Qualitätsmanagementhandbuch (QMH):** übergeordnetes Dokument, welches das Qualitätsmanagement einer Organisation festlegt
- **Qualitätsziel:** Zu erreichendes Ergebnis bezüglich Qualität

4 Allgemeine Anforderungen

4.1 Unparteilichkeit

	Forderung	Ziel der Forderung	Umsetzung
4.1.1	Die Labortätigkeiten werden unparteilich durchgeführt.	Die Forschungsergebnisse sollen nicht beeinflusst oder den Eindruck machen, dass sie beeinflusst werden.<br>Objektivität und Interessenskonfliktdarlegung ist eine Voraussetzung für eine unverzerrte Einordnung und Interpretation der Forschungsergebnisse durch andere Wissenschaftler.<br><br>Risiken für Unparteilichkeit in der Forschung liegen vor allem bei Fördermittelgebern, die eine Erwartung an die Forschungsergebnisse haben, sowie Arbeitgeber in der Industrie (z.B. Pharmafirmen), die Forschungsergebnisse für die Umsetzung neuer Produkte benötigen. Wenn das Ergebnis wichtig ist, um weitere Mittel zu bekommen oder publizieren zu können.	Das Labor analysiert regelmäßig, ob die Unparteilichkeit gefährdet werden könnte. Das Personal wird regelmäßig zum Thema Unparteilichkeit geschult.<br><br>**Vorgehen für Dokumentation:**<br>• Beschreiben der Unparteilichkeit in einem zentralen Dokument (z.B. Qualitätsmanagement-Handbuch oder ein anderes Dokument der Organisation)<br>• Dokument über Korruptionsprävention<br>• Schulung der Mitarbeiter<br>• Regelmäßige Unparteilichkeitsanalyse in Zusammenhang mit der allgemeinen Risikobetrachtung für das Forschungslabor
4.1.2	Die Leitung des Laboratoriums verpflichtet sich zur Unparteilichkeit.		
4.1.3	Das Laboratorium ist für die Unparteilichkeit seiner Labortätigkeiten verantwortlich sein. Es darf keinen kommerziellen, finanziellen oder sonstigen Druck zulassen, der die Unparteilichkeit gefährdet.		
4.1.4	Das Laboratorium identifiziert laufend die Risiken für seine Unparteilichkeit. Hierzu werden auch solche Risiken einbezogen, die aus seinen Tätigkeiten, aus seinen Beziehungen oder aus den Beziehungen seines Personals entstehen.		
4.1.5	Wird ein Risiko für die Unparteilichkeit identifiziert, so beseitigt oder minimiert das Laboratorium dieses Risiko.		

Wird die **Unparteilichkeit** sichergestellt?
Werden die **Interessenskonflikte** offen darlegt und geklärt?
Werden **Risiken für die Unparteilichkeit identifiziert** und diesbezüglich Maßnahmen ergriffen?

Erfüllt? ☐

4.2 Vertraulichkeit

	Forderung	Ziel der Forderung	Umsetzung
**4.2.1**	Das Labor trägt Verantwortung für die Handhabung aller Informationen, die aus Labortätigkeiten erhalten werden. Die Vertraulichkeit von Angaben wird eingehalten.	Gewährleistung der Vertraulichkeit und des Datenschutzes. Das Forschungslabor gewährleistet den Schutz der vertraulichen Informationen und Eigentumsrechte. Erfüllung ethischer, vertraglicher und gesetzlicher Anforderungen.	Das Labor untersucht, ob die Vertraulichkeit und Vorgaben des Datenschutzes sowohl intern als auch extern eingehalten werden. Das Personal wird zu den Themen Vertraulichkeit und Datenschutz regelmäßig geschult.<br><br>**Vorgehen für Dokumentation:**<br>• Beschreiben der Vertraulichkeit in einem zentralen Dokument (z.B. Qualitätsmanagement-Handbuch oder ein anderes Dokument der Organisation)<br>• Verfassen einer Datenschutzleitlinie<br><br>Werden **Vertraulichkeit und Datenschutz** sichergestellt?
**4.2.2**	Das Personal, einschließlich Gremienmitglieder, Vertragspartner, Personal aus externen Stellen oder Personen, die im Auftrag des Laboratoriums tätig sind, behandelt alle Informationen, die es während der Durchführung der Labortätigkeiten erhalten oder geschaffen hat, vertraulich, außer es ist gesetzlich zu anderem verpflichtet.		

Erfüllt? ☐

4.3 Bestimmung des Kontexts

	Forderung	Ziel der Forderung	Umsetzung
	Die Forschungstätigkeiten werden so ausgeführt, dass die Anforderungen der Aufsichtsbehörden, der Organisation, der Kunden und ggf. weiterer interessierter Parteien erfüllt werden. Die Anforderungen werden überwacht.	Anforderungen an das Labor müssen eingehalten werden (Bsp.: regulatorische Zulassungen, im Hinblick auf Gentechnik und Umgangsgenehmigungen mit Biostoffen).	Kontinuierliche Überwachung der geltenden Bestimmungen und Umsetzung von Änderungen. Erwartungen an das Labor werden analysiert und wenn möglich umgesetzt.<br>Gesetzestexte und Verordnungen werden gesammelt und an einem Ort zum Nachlesen aufbewahrt. Das Personal wird geschult.

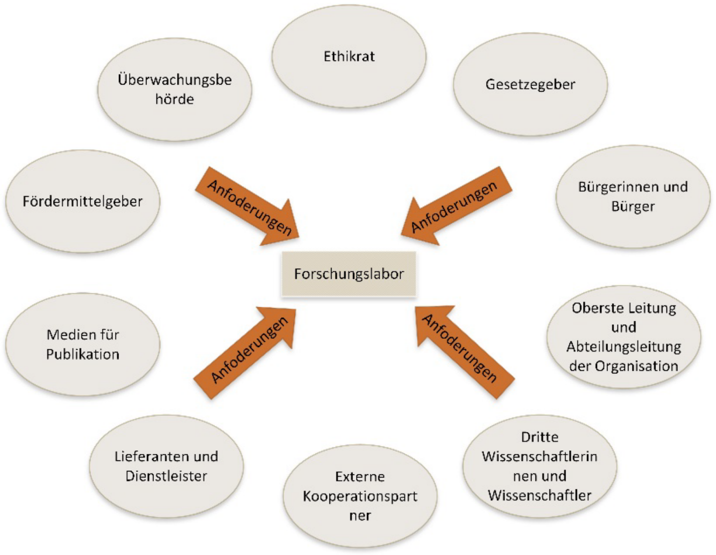

Abbildung 1 Beispiele interessierter Parteien eines Forschungslabors

Werden die geltenden **Gesetze, Regularien und Anforderungen** an das Labor überwacht und sind diese zum Nachlesen an einem Ort gesammelt?

Erfüllt? ☐

4.4 Verpflichtung zur Guten Wissenschaftlichen Praxis

	Forderung	Ziel der Forderung	Umsetzung
**4.4.1**	Die Leitlinien der Guten Wissenschaftlichen Praxis sind zu jederzeit zu befolgen. Sie bilden die Grundlage dieses Dokumentes.<br>Die Niederschrift der Guten Wissenschaftlichen Praxis ist bekannt und zugänglich für das Laboratorium (GWP 1).	GWP Vorgabe als allgemeingültige Basis soll bekannt sein und erfüllt werden. Die Einhaltung der GWP ist unter anderen eine externe Erwartung an ein Forschungslabor, welche keine gesetzliche oder behördliche Anforderung ist (s. 4.3).	• Sicherstellung, dass alle Mitarbeiter bzgl. der GWP geschult sind<br>• Ombudsperson und Verhalten bei Verdacht von wissenschaftlichen Fehlerverhalten müssen bekannt sein<br><br>**Vorgehen für Dokumentation**
**4.4.2**	Eine Ombudsperson mit Vertretung muss existieren und bekannt gemacht sein (GWP 6).		

**4.4.3**	Das Verhalten bei Verdacht von wissenschaftlichen Fehlverhalten muss definiert und für jeden zugänglich sein. (GWP 18,19)		• Verfassen einer Richtlinie zur Einhaltung der GWP in der Organisation<br>• Benennung von GWP- Beauftragten/Ombudsperson<br><br>Wird die **GWP** eingehalten?<br>Gibt es eine **Ombudsperson**?<br>Gibt es einen Prozess für Verdacht auf **wissenschaftliches Fehlverhalten**?<br><br>**Erfüllt? ☐**

4.5 Ethisches Verhalten

	Forderung	Ziel der Forderung	Umsetzung
**4.5.1**	Das Labor hat geeignete Verfahren, um sicherzustellen, dass die Belegschaft menschliche oder tierische Proben, Gewebe oder sterbliche Überreste entsprechend der einschlägigen gesetzlichen Bestimmungen behandelt.	Gewährleistung ethischen Verhaltens bei Umgang mit Proben (auch u.a. eine Erwartung nach 4.3.).	• Einhaltung der gesetzlichen Bestimmungen, Einholung von Ethikvoten und Umsetzung derer Anforderungen<br><br>**Vorgehen für Dokumentation**<br>• Stellungnahme im Qualitätsmanagementhandbuch oder einem anderen zentralen Dokument formulieren<br>• Archivierung der Ethikvoten<br><br>Ist **ethisches Verhalten** in meiner Arbeitsgruppe sichergestellt?<br><br>**Erfüllt? ☐**
**4.5.2**	**Dual Use**<br>Es ist eine gründliche Abschätzung der Forschungsfolgen und Beurteilung der jeweiligen ethischen Aspekte des Forschungsthemas vorzunehmen. (GWP 10)	Sicherstellung, dass die Sicherheit und mögliche mehrfache Verwendbarkeit der Forschung bedacht wurde. Vermeidung von missbräuchlicher Verwendung der Forschungsergebnisse.	Kontinuierliche Bewertung der Dual Use eines Forschungsvorhabens durch einen standardisierten Prozess.<br><br>**Vorgehen für Dokumentation**<br>• Etablierung eines standardisierten Prozesses zum Umgang mit Dual-Use (Formblatt zur Bewertung erstellen)

Wird die Dual Use vor Forschungsbeginn bewertet und bei unerwarteten Entwicklungen die Bewertung angepasst?

Erfüllt? ☐

5 Strukturelle Anforderungen

5.1 Rechtsträger

	Forderung	Ziel der Forderung	Umsetzung
	Das Laboratorium oder die Organisation, zu der das Laboratorium gehört, muss eine Einheit sein, die für ihre Tätigkeiten rechtlich verantwortlich ist.	Verantwortung für Handeln tragen (haftungsrelevante Fragestellungen).	In der Regel ist dieser Punkt bereits erfüllt.<br><br>Ist das Labor eine Einheit einer **rechtlich verantwortlichen** Organisation?<br><br>**Erfüllt? ☐**

5.2 Laborleiter

	Forderung	Ziel der Forderung	Umsetzung
	Das Labor muss eine Leitung benennen, die die Gesamtverantwortung trägt.	Eine Person hat die Gesamtverantwortung für die Organisation und das Handeln des Labors.	In der Regel ist dieser Punkt bereits erfüllt.<br>Eine Leitung des Labors ist schriftlich benannt.<br><br>Gibt es eine **verantwortliche Person** für die Leitung des gesamten Labors?<br><br>**Erfüllt? ☐**

5.3 Organisationsstrukturen

	Forderung	Ziel der Forderung	Umsetzung
5.3.1	Das Laboratorium:<br>a) Beschreibt die Organisation und Managementstruktur des Laboratoriums und dessen Stellung in einer gegebenenfalls vorhandenen Dachorganisation<br>b) Legt die Verantwortlichkeiten, Befugnisse und Wechselbeziehungen des gesamten Personals fest, welches Arbeiten anleitet,	Strukturen, Aufgaben, Prozesse und Kommunikationswege müssen bekannt sein sowohl in der gesamten Organisation als auch in dem einzelnen Forschungslabor. Das unterstützt vor allem neue Mitarbeitende sich schnell in dem	Organisationsstrukturen und Aufgaben in einem Organigramm verdeutlichen. Verantwortlichkeitsübersicht und Autorisierungsplan als Kompetenzübersicht für das Personal erstellen.<br><br>**Vorgehen für Dokumentation**<br>• Organigramm erstellen<br>• Autorisierungsplan/ Kompetenzübersicht erstellen

	Forderung	Ziel der Forderung	Umsetzung
	durchführt oder verifiziert, die die Forschungsergebnisse beeinflussen	Forschungslabor zurechtzufinden und Ansprechpartner zu kennen.	• Verantwortlichkeitsübersicht erstellen<br>Gibt es einen **Autorisierungsplan oder eine Kompetenzübersicht und** eine Verantwortlichkeitenübersicht?<br>Gibt es ein **Organigramm** für das Forschungslabor und die gesamte Organisation?<br>**Erfüllt? ☐**
5.3.2	Die Verfahren werden in dem Umfang dokumentiert, der erforderlich ist, um die konsistente Anwendung der Labortätigkeiten und die Validität der Ergebnisse sicherzustellen.		

5.4 Anforderungen an die Leitung der Organisation

	Forderung	Ziel der Forderung	Umsetzung
5.4.1	Die oberste Leitung trägt die Gesamtverantwortung für die Wirksamkeit des QMS in der Organisation.	Ohne die Einbindung der obersten Leitung in das QMS der Organisation, hat das QM-System kein Rückhalt und wird nicht wertschöpfend funktionieren.<br><br>Die oberste Leitung bestimmt die Arbeitsweise und die Ausrichtung (Vision, Mission, Werte, Kultur) des Unternehmens. Sie ist verantwortlich für die Arbeitsfähigkeit der Organisation. Sie ermöglicht ein funktionierendes QMS durch Ressourcenbereitstellung und klare Definition von Befugnissen und Verantwortlichkeiten.	Das Labor ist Teil des QMS der Organisation (wenn vorhanden) und agiert nach den übergeordneten Organisationsanforderungen.<br><br>Schafft die Leitung Ihrer Organisation die **Rahmenbedingungen** für wissenschaftliches Arbeiten?<br>Wurde die **GWP** in Ihrer Organisation etabliert?<br>**Erfüllt? ☐**
5.4.2	Sie schafft die Rahmenbedingungen für wissenschaftliches Arbeiten einschließlich festgelegter Verfahren und Grundsätze zur Personalauswahl und – Entwicklung sowie zur Förderung des wissenschaftlichen Nachwuchses und der Chancengleichheit *(GWP 3).*		
5.4.3	Sie ist zuständig für Einhaltung und Vermittlung der GWP *(GWP 2, 3).*		

5.5 Anforderungen an die Leitung des Labors

	Forderung	Ziel der Forderung	Umsetzung
5.5.1	Die Laborleitung ist zuständig für:		**Vorgehen für Dokumentation**

	Forderung	Ziel der Forderung	Umsetzung
	a) Organisationsverantwortung;<br>b) Kompetenzvermittlung;<br>c) wissenschaftliche Begleitung;<br>d) Aufsichts- und Betreuungspflichten (GWP 4).	Definition der allgemeinen Aufgaben der Laborleitung gemäß GWP. Ohne eine gute Führung existieren keine motivierten, engagierten und kreativen Mitarbeiter.	• Job Dokument, in dem die Anforderungen und Aufgaben an die Leitungsposition beschrieben wird, erstellen<br>• Formblatt für Betreuung von wissenschaftlichem Nachwuchs erstellen
5.5.2	Sie ist verantwortlich für die Betreuung des wissenschaftlichen Nachwuchses und die Karriereförderung von wissenschaftlichem und wissenschaftsakzessorischem Personal (GWP 4).		Gibt es eine „JOB Beschreibung“ für die Laborleitung unter Einbeziehung folgender Forderungen:<br>• Organisationsverantwortung<br>• Kompetenzvermittlung<br>• wissenschaftliche Begleitung<br>• Aufsichts- und Betreuungspflichten<br>Erfolgt eine systematische Betreuung des **wissenschaftlichen Nachwuchses** durch den Laborleiter?
5.5.3	Die Laborleitung legt Verantwortlichkeiten und Befugnisse fest, dokumentiert und kommuniziert diese.		
			**Erfüllt? ☐**

5.6 Qualitätsmanagement

	Forderung	Ziel der Forderung	Umsetzung
5.6.1	Das Labor baut für die Forschungsaktivitäten ein gemäß seiner Größe passendes QMS auf, unterhält und entwickelt es weiter. Dabei ist folgendes zu beachten:<br>a) Einbeziehung aller qualitätsrelevanten Prozesse<br>b) Sämtliche Dokumentationen, Prozesse, Systeme, Aufzeichnungen, die sich auf die Erfüllung der Anforderungen dieses Dokuments beziehen werden ins Managementsystem eingebunden<br>c) Personal muss Zugang zu den Dokumentationen haben, die unter ihre Verantwortung fallen	Standardanforderung (s. ISO/IEC 17025 8.1.1, ISO 15189 8.1.1, ISO 9001 4.4.1).<br>Der Anforderungskatalog beschreibt nicht, wie ein QMS aussehen muss, sondern gibt nur einen Rahmen vor, sodass das QMS für jede Forschungslaborgröße und -ausstattung gestaltbar ist.	Qualitätsrelevante Prozesse der Organisation (Bspw. Beschaffung, Feedback, Risiko und Chancen, Probeneingang) und des Forschungslabors (Bspw. Probenahme, Resistenztestung, Medien, S3 An-/Auskleiden) sind nachvollziehbar zu dokumentieren. Ziel: Personal kann Prozesse nachlesen, Archivierung für Nachvollziehbarkeit auch noch in vielen Jahren, Laborleiter hat Kontrolle über Prozesse.<br><br>**Vorgehen für Dokumentation**<br>• Verfahrensanweisungen für Standardprozesse erstellen und lenken

			Gibt es **Anweisungen und Aufzeichnungen** für qualitätsrelevante Prozesse?<br>Hat das Personal **Zugang zu den Verfahrensanweisungen, Arbeitsanweisungen** etc., die unter ihre Verantwortung fallen?<br>**Erfüllt? ☐**
**5.6.2**	Das Laboratorium verfügt über Personal, das, ungeachtet seiner anderen Verantwortlichkeiten, über die erforderlichen Befugnisse und Ressourcen verfügt, um den Aufgaben für Pflege, Erhalt und Weiterentwicklung des Qualitätsmanagements nachzukommen.	Eine für das QM-System Hauptverantwortliche Person und Ansprechpartner. Zu beachten: nicht er alleine etabliert und kümmert sich um das QMS. Alle Labormitarbeiter arbeiten mit und leben es (SOPs schreiben, Abweichungen dokumentieren, etc.). Verantwortlich ist die Laborleitung für die Umsetzung des QMS, nicht der Qualitätsmanagementbeauftragte.	Benennung einer oder mehrerer verantwortlicher Personen für das QMS (CAVE: die Person benötigt auch Zeit für seine Arbeit am QMS).<br><br>**Vorgehen für Dokumentation**<br>• QMB offiziell bestimmen und im Organigramm und Verantwortlichkeitsübersicht eintragen<br><br>Besitzt das Labor eine oder mehrere Personen die zuständig für das Qualitätsmanagement im Labor sind (**Qualitätsmanagementbeauftragter**)?<br>**Erfüllt? ☐**
**5.6.3**	Die oberste Leitung definiert den Zweck des Forschungs-QMS in der Qualitätspolitik.	Die Qualitätspolitik erwächst aus der Vision und Mission der Organisation, und ist somit die strategische Ausrichtung in Bezug auf Qualität. Sie stellt den übergeordneten Rahmen für die spezifischeren Qualitätsziele und der daraus abgeleiteten Maßnahmen dar. Die Normenreihe DIN EN ISO 9000 definiert Qualitätspolitik als eine Erklärung übergeordneter Absichten und Ausrichtung einer Organisation zur Qualität.	Durch die <u>oberste Leitung</u> wird eine Qualitätspolitik definiert, die im Einklang mit den Zielen der Organisation stehen:<br>• Zweck und Kontext der Organisation angemessen<br>• Rahmen zur Festlegung von Q-Zielen<br>• Verpflichtung zur fortlaufenden Verbesserung des QMS<br><br>Gibt es eine **Qualitätspolitik** der Organisation?<br>Wurde das Forschungs-QMS in dieser mit aufgenommen?<br>**Erfüllt? ☐**

6 Planung der Forschungsprozesse

6.1 Ziele

Forderung	Ziel der Forderung	Umsetzung
Das Labormanagement legt Qualitätsziele fest, überwacht und, kommuniziert sie und passt sie falls erforderlich an. Die Qualitätsziele sind messbar und im Einklang mit der Qualitätspolitik.	Definition der Ziele für das Forschungslabor durch die Laborleitung.<br>Planung eines Forschungsjahres: Was will ich mit meinem Team erreichen? Welche Herausforderungen und Chancen gibt es? Wichtig bei mehreren Projekten gleichzeitig. Eine Definition von Zielen erzeugt einen roten Faden für das Forschungsjahr und hilft bei eventuellen Priorisierungen.	Qualitätsziele für das Forschungslabor festlegen (s. Abb.2).<br><br>**Vorgehen für Dokumentation**<br>• Qualitätsziele, Zeitraum, Zuständigkeit dokumentieren<br><br>Wurden **Qualitätsziele für das Labor** bestimmt und kommuniziert (nach einem Jahr prüfen, ob erfüllt und neue formulieren)?<br><br>**Erfüllt? ☐**

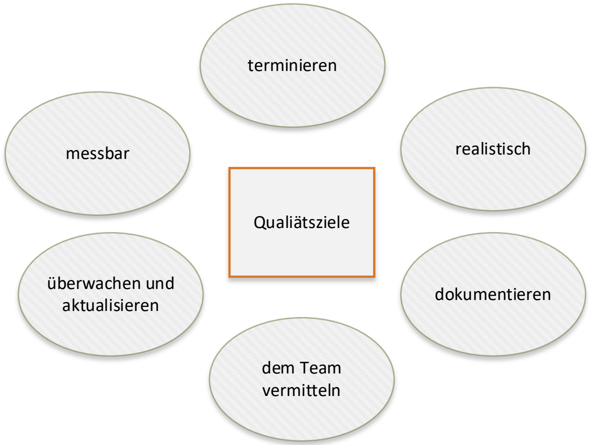

Abbildung 2 Qualitätsziele

6.2 Maßnahmen zum Umgang mit Risiken und Chancen

	Forderung	Ziel der Forderung	Umsetzung
**6.2.1**	Sowohl laborinterne als auch externe Risiken und Chancen für den Erfolg des Forschungsprojekts sind bei der Forschungsplanung zu berücksichtigen, um:<br><br>a) Prozesse zu verändern, und Risken zu verringern/beseitigen;	Anstelle einer nachträglichen Fehlererkennung werden qualitätsrelevante Risiken bereits im Voraus identifiziert und vorgebeugt. Eine Ermittlung von Risiken und Chancen kann von Anfang an Ressourcen,	Überlegungen zu möglichen Risikoquellen machen (interne Themen, externe Themen, interessierte Parteien) und Chancen für seine Forschung identifizieren.<br><br>**Vorgehen für Dokumentation**<br>Formblatt für die Sammlung und Analyse von Risiken erstellen

	b) die Chancen zu erhöhen, die Absichten und Ziele des Laboratoriums zu erreichen;<br>c) Potenzielle Fehler im Voraus zu identifizieren und potenzielle Störungen für die Ergebnisse zu bewerten;<br>d) unerwünschte Auswirkungen und Ausfälle bei den Labortätigkeiten zu verhindern oder zu reduzieren.	Zeit und Kosten sparen und Fehler vermeiden.<br>Erreichen von gewünschten Zielsetzungen, Verstärkung erwünschter Auswirkungen, verhindern oder verringern unerwünschter Auswirkungen.<br>Durch eine Dokumentation von identifizierten Risiken werden diese für spätere Risikobetrachtungen bereits aufgeführt und müssen nur im neuen Kontext bewertet werden.<br><br>CAVE: Fehler können etwas Gutes in der Forschung sein, die Nichterfüllung des Forschungsziels aufgrund von vermeidbaren Komplikationen nicht.	Werden **Risiken und Chancen** des Forschungslabors/ der Forschungsprojekte bewertet?<br>Werden Maßnahmen diesbezüglich eingeleitet?<br><br>**Erfüllt? ☐**
**6.2.2**	Die Maßnahmen zum Umgang mit Risiken und Chancen müssen proportional sein.		
**6.2.3**	Das Laboratorium zeichnet die Risiken sowie die getroffenen Entscheidungen und Maßnahmen auf.		

7 Anforderungen an Ressourcen

7.1 Allgemeines

	Forderung	Ziel der Forderung	Umsetzung
	Für die Durchführung der Labortätigkeiten verfügt das Labor über Personal, Räumlichkeiten, Einrichtungen, Systeme und unterstützende Dienstleistungen.	Labor muss fähig und kompetent sein, die Untersuchungsverfahren für das Forschungsprojekt durchzuführen.	**Erfüllt? ☐**

7.2 Personal

	Forderung	Ziel der Forderung	Umsetzung
7.2.1	Die Leitung teilt dem Personal Pflichten, Verantwortlichkeiten und Befugnisse mit.	Klare Verantwortlichkeiten und Befugnisse sollen Missverständnisse und damit Fehler und Zeitverlust vermeiden. Eine klare	Laborleitung ordnet klar Verantwortlichkeiten seinem Laborteam zu. CAVE: Hat die Person die notwendigen Ausbildungen und Schulungen? Die Kompetenzen der Mitarbeitenden werden in einem Autorisierungsplan / einer Kompetenzübersicht festgehalten.

		Kommunikation von Aufgaben und Befugnissen erleichtert den Arbeitsalltag.<br>Durch einen Autorisierungsplan/ eine Kompetenzübersicht erhalten Mitarbeiter eine Übersicht über die Methodenkompetenzen die im Labor vorhanden sind.	**Vorgehen für Dokumentation**<br>Entwurf von Autorisierungsplan / Kompetenzübersicht und Verantwortlichkeitsübersicht<br><br>Gibt es eine Verantwortlichkeitsübersicht und einen **Autorisierungsplan / Kompetenzübersicht**?<br><br>**Erfüllt? ☐**
7.2.2	Neue Mitarbeiter werden eingearbeitet.<br>Es erfolgt ein Wissenstransfer bei ausscheidenden Mitarbeitern.	Eine einheitliche, systematische Einarbeitung dient der schnellen und gezielten Wissensweitergabe. Eine gute Einarbeitung der Mitarbeiter verkürzt die Zeit bis ein neuer Kollege Tätigkeiten selbstständig sicher übernehmen. Der Wissenstransfer beim Ausscheiden ist wichtig, damit das Wissen (Bspw. Methoden, Geräte, spezielle Verfahren) bei ausscheidenden Mitarbeitern nicht verloren geht. Anderenfalls muss es von Neuem angeeignet werden, was wiederum Zeit und Ressourcen kostet. Eine Fehlerminimierung und Effizienzsteigerung ist das Ziel.	Prozess für Einarbeitung und Ausscheiden von Mitarbeitern systematisieren. Wissenssicherung von ausscheidenden Mitarbeitern durch Verschriftlichung von Wissen in Form von Arbeitsanweisungen, direkte Übergabe von Dokumenten, Ergebnisse und Aufzeichnungen an Teammitglieder, direkte Einarbeitung und Übergabe von neuen Mitarbeitern durch den ausscheidenden Kollegen.<br><br>**Vorgehen für Dokumentation**<br>Erstellung von einem Einarbeitungsplan und -dokumentation und einer Verabschiedungscheckliste<br><br>Gibt es einen **Einarbeitungsplan** für neue Mitarbeiter?<br>Gibt es eine **Wissenssicherung** bei ausscheidenden Mitarbeitern?<br><br>**Erfüllt? ☐**
7.2.3	Eine Unterweisung und Verpflichtung des Personals zum unparteilichen Handeln und zur Geheimhaltung erfolgt.	Verpflichtung zum unparteilichen Handeln und zur Geheimhaltung sind Grundvoraussetzungen für ein vertrauensvolles Umfeld (Voraussetzung für Erfüllung 4.1 und 4.2) und seriöse Forschung.	Erfolgt meist in Organisationen durch Unterzeichnung der Arbeitsverträge.<br><br>**Erfüllt? ☐**

7.2.4	Das Labor stellt sicher, dass die Mitarbeiter über notwendige Kompetenz verfügen.<br>Es werden regelmäßig Qualifizierungsmaßnahmen, Schulungen und Fortbildungen besucht. Eine Teilnahme an GWP-Schulungen (GWP 2) ist Pflicht. Die jeweiligen Schulungsziele der Mitarbeiter werden mit dem Leiter kommuniziert (z.B. im Mitarbeitergespräch). Im Anschluss an entsprechende Schulungen wird die Wirksamkeit dieser bewertet.	Mitarbeiter sollen für die Tätigkeiten, die sie ausführen, kompetent sein. Das minimiert die Fehlerquote. Das Personal ist ein wichtiger Einflussfaktor für die Qualität der Forschung.<br><br>Beispiele für mögliche Schulungsfelder:<br>• relevante Kenntnisse und Fähigkeiten<br>• Geräte<br>• Gesetze, Verordnungen, Normen, Richtlinien<br>• Soziale Kompetenz	Regelmäßig evaluieren, welcher Mitarbeiter, welche Schulung besuchen muss. Übersicht über Schulungsteilnahme führen und Zertifikate sammeln. Kompetenzanforderungen in Arbeitsplatzbeschreibungen (JOB-Dokumente) vorhanden.<br><br>**Vorgehen für Dokumentation**<br>Schulungsübersicht, Zertifikate sammeln, Dokumentation der Schulungsziele bei Mitarbeitergesprächen<br><br>Gibt es eine **Schulungsübersicht**, welche Mitarbeiter, welche Schulungen besucht haben?<br>Welche Mitarbeiter benötigen noch welche Schulungen?<br><br>**Erfüllt?** ☐
7.2.5	Es gibt Tätigkeitsbeschreibungen inkl. Kompetenzanforderung an die jeweilige Position für jeden Arbeitsbereich.<br><br>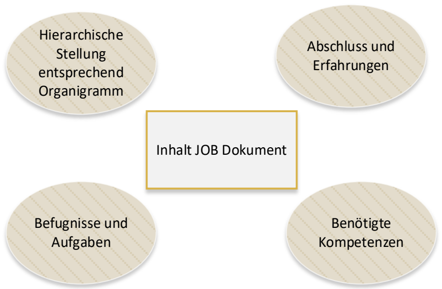<br><br>*Abbildung 3 Inhalt JOB Dokument*	Mit einer Stellenbeschreibung entscheidet der Laborleiter über die Aufgaben und Befugnisse einer Position und bestimmt welche Kompetenzen und Erfahrungen dafür notwendig sind.	**Vorgehen für Dokumentation**<br>Arbeitsplatzbeschreibungen anfertigen<br><br>Gibt es Stellenbeschreibungen (JOB Dokument) mit festgelegten Kompetenzanforderungen für die vorhandenen Positionen im Labor?<br><br>**Erfüllt?** ☐
7.2.6	Es werden Aufzeichnungen unterhalten über maßgebliche Abschlüsse, Fortbildungen, Erfahrungen und Beurteilungen der Kompetenz von Mitarbeitern.	Allgemeine Anforderung nach Personalakte	Diese Forderung wird meist durch die Personalabteilung erfüllt.<br><br>**Erfüllt?** ☐

7.2.7	Eine Bewertung der Leistung des Personals (s. GWP 5) findet in regelmäßigen Abständen statt. Die persönliche Zielsetzung und die Zufriedenheit und Wünsche am Arbeitsplatz werden besprochen.	Förderung der Zusammenarbeit, gegenseitige Feedbackgabe, Besprechung von Entwicklungswünschen stärkt das Führungskraft-Mitarbeiter-Verhältnis. Klare Kommunikation von Erwartungen und Wünschen mindert Missverständnisse.	Durchführung und Dokumentation von regelmäßigen Mitarbeitergesprächen. Die Gespräche verlaufen in gegenseitigem Respekt und der Inhalt und die Vereinbarungen sind streng vertraulich.<br><br>**Vorgehen für Dokumentation:**<br>In der Regel gibt es bereits festgelegte Vorgehensweisen für Mitarbeitergespräche durch die jeweilige Organisation. Falls das nicht der Fall sein sollte, wird ein Prozess für diese im Labor etabliert und dokumentiert.<br><br>Werden **Mitarbeitergespräche** regelmäßig durchgeführt?<br><br>**Erfüllt? ☐**

7.3 Kommunikation

	Forderung	Ziel der Forderung	Umsetzung
7.3.1	Rollen und Verantwortlichkeiten der an einem Forschungsvorhaben beteiligten Akteure müssen zu jedem Zeitpunkt des Vorhabens klar sein. Die Rollen sind anpassbar nach Absprache. (GWP 8)	Durch eine klare Kommunikation von Rollen und Verantwortlichkeiten werden Missverständnisse (Doppelarbeiten, Fehler, etc.) vermieden.<br>Durch Besprechungsprotokolle können mündliche Verabredungen und Aufgabenzuweisungen nachgelesen werden. Mitarbeitende, die bei der Besprechung nicht anwesend sind, können sich informieren.	Rollen und Verantwortlichkeiten eines Projekts werden in einer Übersicht für alle einsehbar geführt. Wenn Verantwortlichkeiten angepasst werden, wird die Übersicht aktualisiert. Protokolle von Besprechungen führen und an einem Ort für alle Teammitglieder zugänglich ablegen.<br><br>**Vorgehen für Dokumentation**<br>Übersicht über Verantwortlichkeiten eines Projekts führen und Besprechungen protokollieren
7.3.2	Die Beteiligten eines Forschungsvorhabens stehen in einem regelmäßigen Austausch und legen ihre Rollen und Verantwortlichkeiten in geeigneter Weise fest, und passen diese, sofern erforderlich, an. (GWP 8)		
7.3.3	Es erfolgt eine Einbindung der Projektmitarbeiter in die für das Projekt vorgesehenen Kommunikationswege (Meetings, Netzlaufwerke, E-Mailverteiler).		

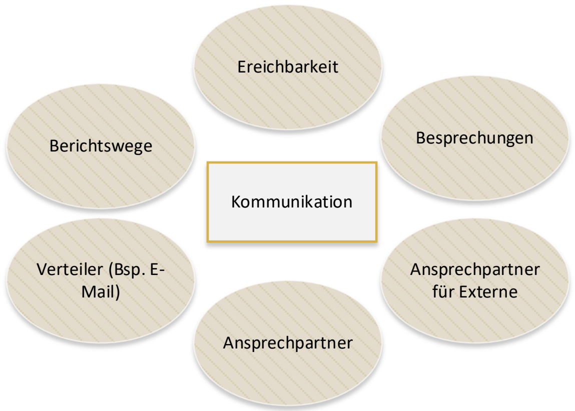

Abbildung 4 Kommunikation

Sind **Rollen und Verantwortlichkeiten** im Forschungsprojekt festlegt und kommuniziert?
Sind die Zuständigkeiten in dem jeweiligen Projekt klar?
Ist das Personal in alle notwendigen **Kommunikationswege** eingebunden?
Werden **Protokolle** bei Labormeetings geschrieben und nach dem Meeting verteilt?

Erfüllt? ☐

7.4 Räumlichkeiten und Umgebungsbedingungen

	Forderung	Ziel der Forderung	Umsetzung
7.4.1	Räumlichkeiten und Umgebungsbedingungen sind für die Tätigkeiten geeignet. Sowohl die Validität der Ergebnisse (Einflüsse z.B. mikrobielle Verunreinigungen, Staub, elektromagnetische Störungen, Strahlung, Feuchtigkeit, elektrische Energieversorgung, Temperatur, Schall und Vibrationen) als auch die Sicherheit des Personals dürfen nicht nachteilig beeinflusst werden.	Räume und Umwelteinflüsse dürfen die Validität der Ergebnisse nicht beeinflussen. Personen dürfen nicht gefährdet werden.	Räume müssen auf Angemessenheit und Zweckmäßigkeit geprüft werden. Relevante Umgebungsbedingungen für die Versuche müssen bekannt sein.<br><br>**Vorgehen für Dokumentation:**<br>• In der Regel durch die Organisation geregelt<br>• Falls nicht: Dokumentation der Prüfung der Räumlichkeiten auf Angemessenheit, Zweckmäßigkeit und Arbeitsschutz<br><br>**Erfüllt? ☐**
7.4.2	Das Laboratorium legt die Angemessenheit und Zweckmäßigkeit des Raumes für die jeweilige Labortätigkeit fest, bewertet und stellt diese sicher.	Der Raum muss auf Tauglichkeit geprüft sein.	
7.4.3	Relevante Umgebungsbedingungen (Licht, Sterilität, Staub, giftige oder gefährliche Dämpfe, elektromagnetische Störungen,	Einfluss von Umgebungsbedingungen auf die Qualität der Forschung und Validität der Ergebnisse	**Vorgehen für Dokumentation:**<br>Relevante Umgebungsbedingungen überwachen und dokumentieren.

	Strahlung, Feuchtigkeit, Energieversorgung, Temperatur, Schall- und Schwingungspegel und Logistik des Arbeitsablaufs) werden überwacht und aufgezeichnet.	überwachen, Arbeitssicherheit gewährleisten.	Werden die **Umgebungsbedingungen** überwacht, die sich auf die Proben und Methoden auswirken könnten?<br><br>**Erfüllt? ☐**
7.4.4	Eine Instandhaltung der Räumlichkeiten erfolgt.<br>Die Funktionsweise des Raumes wird regelmäßig überprüft (Wartungen).	Regelmäßige Reinigung, Wartungen, Arbeitsschutz	Regelmäßige Reinigung und Wartung der Räume.<br><br>**Vorgehen für Dokumentation:**<br>• In der Regel über die Organisation gesteuert und dokumentiert.<br>• Falls nicht: Wartungsplan und -dokumentation, sowie Reinigungsdokumentation erstellen.<br><br>Erfolgt eine regelmäßige **Instandhaltung und Wartung** der Laborräume?<br><br>**Erfüllt? ☐**
7.4.5	Der Zugang zu den Bereichen wird geregelt, sodass ein Schutz vor unbefugtem Zugriff vorhanden ist.	Nur befugte Personen haben Zutritt zu den Räumlichkeiten (Schutz des Projektes, der Geräte, sensibler Daten).	Überwachen des Zugangs zu Räumlichkeiten. Zugangsberechtigungen aktuell halten.<br><br>**Vorgehen für Dokumentation:**<br>• Übersicht führen, wer Zugangsberechtigung zu den Räumen hat und aktuell halten. In der „Weggangscheckliste“ für ausscheidendes Personal den Punkt „Zugangsberechtigung entfernen“ aufnehmen<br><br>Welche Personen haben **Zugang zu Räumlichkeiten**?<br>Werden Personen, die keinen Zugang mehr brauchen, auch aus der **Berechtigung entfernt**?<br><br>**Erfüllt? ☐**

7.4.6	Trennung von Laborbereichen für miteinander unvereinbare Tätigkeiten und Verhinderung von Kontamination, sowie von Störungen oder nachteiliger Beeinflussung der Labortätigkeiten.	Vermeidung bewusster und unbewusster Kontamination etc.	Räumliche Trennung von unvereinbaren Tätigkeiten, Proben, o.ä. (Arbeitsplätze, Lagerräume, etc.)<br><br>Sind Laborbereiche von miteinander unvereinbaren Tätigkeiten getrennt, sodass **Kontamination** verhindert wird?<br><br>**Erfüllt? ☐**
7.4.7	**Aufbewahrungseinrichtungen**<br>Es existieren Lagerflächen und die notwendigen Bedingungen, die die ständige Unversehrtheit von Proben, Geräten, Material, etc. sicherstellen.	Lagerräume, Schränke, Kühlschränke, etc. müssen geeignet sein, Beeinflussungen des Experiments zu verhindern (Temperatur, Luftfeuchtigkeit, Schädlinge etc.).	Bewertung der Angemessenheit der Aufbewahrungseinrichtungen und wenn relevant Überwachung der Umweltbedingungen innerhalb der Aufbewahrungseinrichtung.<br><br>Wird in den **Aufbewahrungseinrichtungen** auf Kontamination und Lagerbedingungen geachtet?<br><br>**Erfüllt? ☐**

7.5 Ausrüstung

7.5.1 Allgemein

	Forderung	Ziel der Forderung	Umsetzung
7.5.1.1	Das Laboratorium hat Zugang zu Ausrüstungen, die für die korrekte Durchführung der Labortätigkeiten erforderlich sind und welche die Ergebnisse beeinflussen können (z.B. Messgeräte, Software, Referenzmaterialien, Reagenzien, Verbrauchsmaterialien, etc.).	Das Labor ist verantwortlich für seine Geräte (Kalibrierung, Wartung, etc.). Bei fremden Geräten muss überprüft werden, ob diese zuverlässig und richtig messen.	Es muss sichergestellt werden, dass die eigenen und ausgeliehene Geräte richtig messen.<br><br>Werden auch **Geräte** für die Forschung benutzt**, die nicht dem Labor gehören** und zu denen man keinen Zugang hat?<br>Wie stellt man hier sicher, dass diese Geräte richtig messen?<br><br>**Erfüllt? ☐**
7.5.1.2	Falls das Labor Ausrüstungen benutzen muss, die nicht seiner ständigen Kontrolle unterlieg, stellt es die Qualität dieser sicher.		

7.5.2 Anforderungen

	Forderung	Ziel der Forderung	Umsetzung
7.5.2.1	Das Labor verfügt über Verfahren für die Handhabung, den Transport, die Lagerung, den Gebrauch und die vorgesehene Wartung von Geräten (Bspw.: Messgeräte, Software, Normale, Referenzmaterialien, Referenzdaten, Reagenzien und Verbrauchsmaterialien oder Hilfseinrichtungen). Es wird die ordnungsgemäße Funktionsfähigkeit sichergestellt, sowie Kontaminationen und Beeinträchtigungen verhindert.	Es muss bekannt sein, wie mit den Geräten umzugehen ist, damit es nicht zu Schäden oder Messfehlern kommt.	**Vorgehen für Dokumentation:** Arbeitsanweisungen für Geräte erstellen. Gebrauchsanweisungen aufheben.<br><br>Gibt es **Anweisungen für den Umgang mit Geräten**?<br><br>**Erfüllt?** ☐
7.5.2.2	Es ist ein Gerätebetreuer benannt. Dieser trägt die Verantwortung für die Betriebsbereitschaft, Kalibrierung, Wartung, Vorkommnisse der Geräte, Einweisungen und Aufzeichnungen.	Eine Person ist zuständig für ein oder mehrere Geräte. So gibt es einen Ansprechpartner für das Gerät und jemanden der für die Instandhaltung und Wartung, sowie bei auftretenden Problemen zuständig ist. Das verhindert Unstimmigkeiten und Zeitverlust.	Einen Gerätebetreuer für ein oder mehrere Geräte benennen.<br><br>**Vorgehen für Dokumentation:** Zentrale Übersicht über Gerätebetreuer führen und aktuell halten<br><br>Sind **Gerätebetreuer** für Geräte benannt und dokumentiert?<br><br>**Erfüllt?** ☐
7.5.2.3	Einrichtungen erfüllen die festgelegten Anforderungen. Vor Benutzung wird nachgewiesen, dass die Ausrüstungsgegenstände geeignet sind (Annahmeprüfung).	Geräte müssen bei Lieferung auf Funktionstüchtigkeit überprüft werden.	**Vorgehen für Dokumentation:** Annahmeprüfung vor erster Benutzung dokumentieren (z.B. im Geräteordner)<br><br>Werden **Annahmeprüfungen** bei Erhalt von dem Labor für neue Geräte durchgeführt?<br><br>**Erfüllt?** ☐
7.5.2.4	Für die Messungen genutzte Einrichtungen sind geeignet, um ein valides Ergebnis mit einer erforderlichen Messgenauigkeit zu erreichen.	Methoden mit dem richtigen und geeigneten Gerät durchführen.	**Erfüllt?** ☐

7.5.2.5	Geräte werden nur durch geschultes/befugtes Personal bedient. Aktuelle Anweisungen für Handhabung und Gebrauch der Geräte sind leicht verfügbar (Dokumentation Einweisung und Nutzung Geräte).	Unsachgemäßer Gebrauch der Geräte kann zu Verschleiß und zu unbemerkten systematischen Fehlern führen.	**Vorgehen für Dokumentation:**<br>Geräteschulungen in den Einarbeitungsplan aufnehmen und bei Einarbeitung dokumentieren. Eintrag im Autorisierungsplan<br><br>Gibt es **Anweisungen für Geräte**?<br>Werden die Anwender vor Nutzung der Geräte geschult?<br><br>**Erfüllt?** ☐
7.5.2.6	Ein Programm für Wartung und Instandhaltung ist vorhanden. Die Zeitpläne entsprechen mindestens der des Herstellers.<br>Die Ausrüstung ist im Zustand sicherer Arbeitsbedingungen und betriebsfähig.	Geräte müssen gewartet und instandgehalten werden. Fehlerminimierung. Durch Benutzerlisten können, beispielsweise bei im Gerät vergessenen Proben, schnell der richtige Ansprechpartner gefunden werden.	Organisation der Wartung und Instandhaltung der Geräte nach Herstelleranweisung.<br><br>**Vorgehen für Dokumentation:**<br>Wartungsplan und -dokumentation, Benutzerliste führen<br><br>Gibt es **Wartungspläne** und **Nutzerlisten**?<br><br>**Erfüllt?** ☐
7.5.2.7	Geräte, die nicht richtig funktionieren werden außer Betrieb genommen:<br>a) Absondern, um Gebrauch zu verhindern;<br>b) eindeutig kennzeichnen, bis verifiziert wurde, dass sie ordnungsgemäß arbeiten.<br>Das Labor untersucht Auswirkungen des Fehlers auf bereits durchgeführte Versuche.<br><br>Vorkommnisse hinsichtlich der Laboratoriumsausrüstung werden dem Geräteverantwortlichen gemeldet und dokumentiert. Nichtkonformitäten werden den zuständigen Herstellern/Behörden angezeigt.	Messungen mit defekten Geräten verhindern. Wurden dennoch Messungen durchgeführt, muss hieraufgefunden werden, wo die Ergebnisse bereits verwendet wurden, entsprechende Maßnahmen müssen eingeleitet werden.<br>Fehler mit Geräten dokumentieren, damit systematische Fehler frühzeitig erkannt werden.	**Vorgehen für Dokumentation:**<br>Fehler mit Geräten sind in einer Abweichungsliste zu dokumentieren. Entsprechende Maßnahmen einleiten (Wurden die Ergebnisse schon in die Versuchsauswertung einbezogen?).<br><br>Gibt es Anweisung für Verfahren für den Umgang mit **fehlerhaften/defekten Geräten**?<br><br>**Erfüllt?** ☐
7.5.2.8	Für jedes Gerät, das die Laborergebnisse beeinflussen kann, werden Aufzeichnungen geführt (z.B. innerhalb eines Geräteordner). Inhalt:	Überblick über alle Geräte und relevante Informationen zum Gerät. Schnellere Zusammenfassung der Gerätedaten für eine Publikation.	**Vorgehen für Dokumentation:**<br>Geräteordner mit allen relevanten Informationen erstellen.

	Forderung	Ziel der Forderung	Umsetzung
	a) Identitätsbezeichnung der Ausrüstung;<br>b) Name des Herstellers, Gerätetyp und Seriennummer oder eine sonstige eindeutige Identifizierung;<br>c) Kontaktangaben des Lieferanten oder des Herstellers;<br>d) Daten der Lieferung und der Inbetriebnahme;<br>e) Standort;<br>f) Zustand bei Erhalt (z. B. neu, gebraucht oder überholt);<br>g) Anweisungen des Herstellers;<br>h) Aufzeichnungen, die die anfängliche Gebrauchstauglichkeit des Geräts bestätigen, wenn die Ausrüstung in das Laboratorium eingebaut wird;<br>i) die durchgeführte Wartung und der Zeitplan für die vorbeugende Instandhaltung;<br>j) Aufzeichnungen über die Leistungsfähigkeit der Ausrüstung, die die andauernde Gebrauchstauglichkeit des Geräts bestätigt (z.B. Kopien von Berichten/Zertifikaten aller Kalibrierungen);<br>k) Schäden oder Funktionsstörungen, Veränderungen oder Reparaturen des Geräts.	Eine Geräteliste wird unter anderem auch zum Teil von Förderprogrammen und von der Arbeitssicherheit verlangt.	Gibt es einen entsprechenden **Geräteordner** und eine **Übersicht über alle Geräte**?<br><br>**Erfüllt? ☐**

7.6 Kalibrierung der Geräte

	Forderung	Ziel der Forderung	Umsetzung
7.6.1	Messeinrichtungen werden kalibriert, wenn Messgenauigkeit/-ungenauigkeit die Validität der Ergebnisse beeinflusst und /oder	Unkalibrierte Messeinrichtungen können das Ergebnis negativ	**Vorgehen für Dokumentation:**<br>Kalibierverfahren dokumentieren. Zu kalibrierende Geräte kennzeichnen.

	Kalibrierung erforderlich ist, um metrologische Rückführbarkeit der berichteten Ergebnisse herzustellen.	beeinflussen. Unbekannter Fehlerrahmen.	
7.6.2	Ein Programm für die Kalibrierung existiert, welches regelmäßig überprüft und bei Bedarf angepasst wird.		
7.6.3	Einrichtungen, die Kalibrierung erfordern: Dieses Gerät ist gekennzeichnet, beschildert oder anderweitig identifizierbar gemacht (Anwender müssen Kalibrierstatus und Gültigkeitszeitraum leicht erkennen können).		
7.6.4	Inhalt des Kalibrierverfahrens:<br>a) Berücksichtigung der Anwendungsbedingungen und der Gebrauchsanweisung des Herstellers;<br>b) Aufzeichnen der metrologischen Rückverfolgbarkeit des Kalibrierstandards und die rückführbare Kalibrierung des Ausrüstungsgegenstands;<br>c) in festgelegten Abständen eine Überprüfung der geforderten Messgenauigkeit und der Funktionsweise des Messsystems;<br>d) Aufzeichnen des Kalibrierstatus und des Datums der Kalibrierung;<br>e) sicherstellen, dass dort wo die Kalibrierung Anlass zum Einführen von Korrekturfaktoren gibt, die bisherigen Kalibrierfaktoren korrekt aktualisiert werden,<br>f) zur Vorbeugung von Anpassungen oder Verfälschungen, die die Untersuchungsergebnisse ungültig machen können, Sicherheitsmaßnahmen treffen.		Gibt es ein Verfahren für die **Kalibrierung** von Geräten? Werden Geräte die kalibriert sind, **gekennzeichnet** (Kalibrierstatus und Gültigkeitszeitraum)?<br><br>**Erfüllt? ☐**

7.7 Metrologische Rückführbarkeit

	Forderung	Ziel der Forderung	Umsetzung
7.7.1	Die metrologische Rückführbarkeit der Messergebnisse wird mittels einer dokumentierten, ununterbrochenen Kette von Kalibrierungen eingeführt und aufrechterhalten.	Globale Vergleichbarkeit der Werte, Einhaltung von Grenzwerten	Dokumentation<br><br>Sind die Messergebnisse **metrologisch** auf SI-Einheiten **rückführbar**?<br><br>**Erfüllt? ☐**
7.7.2	Die Messergebnisse sind auf SI-Einheiten oder auf andere geeignete Referenzen rückführbar.		
7.7.3	Wenn dies nicht möglich ist, werden andere Mittel zur Vertrauensbildung in die Ergebnisse angewendet, Bsp.:<br>- Verwendung von zertifizierten Referenzmaterialien<br>- Ergebnisse von Referenzmessverfahren, festgelegten Verfahren oder auf Konsens beruhenden Normalen		

7.8 Reagenzien und Verbrauchsmaterial

	Forderung	Ziel der Forderung	Umsetzung
7.8.1	Ein Verfahren für Entgegennahme, Lagerung Annahmeprüfung, Bestandsführung von Reagenzien und Verbrauchsgütern ist existent.	Reagenzien und Verbrauchmaterial haben einen direkten Einfluss auf die Richtigkeit der Ergebnisse. Eine korrekte Handhabung und eine richtige und bekannte Qualität sind für die Interpretation der Ergebnisse wichtig. Falsche Lagerung kann auch bei Reagenzien und Verbrauchsmaterial zu Qualitätsverlusten bis hin zur Unbrauchbarkeit führen.	Verfahren für den Umgang mit Reagenzien einführen. Identität prüfen (Chargenzertifikate aufbewahren).<br><br>**Vorgehen für Dokumentation:**<br>• Führen von Chemikalien-, Material- und Chargenlisten<br>• Dokumentation der Annahme und Leistungsfähigkeit<br>• Bei Chargenwechsel werden "alt und neu" im Vergleich mit Positivkontrollen getestet.
7.8.2	Das Labor lagert nach Angaben des Herstellers.<br><br>Wenn das Labor die Reagenzien/Verbrauchsgüter nicht entgegennimmt, wird geprüft, ob die empfangende Stelle ausreichende Kapazitäten zur Lagerung und Handhabung des Artikels hat.		

7.8.3	Eine Annahmeprüfung erfolgt für jede neue Lieferung. Vor Gebrauch wird auf Leistungsfähigkeit geprüft.		Ist ein Verfahren für **Entgegennahme, Lagerung Annahmeprüfung, Bestandsführung von Reagenzien und Verbrauchsgütern** existent?<br>Erfolgt eine **Annahmeprüfung** für neue Lieferungen?<br>Wird vor Gebrauch auf **Leistungsfähigkeit** geprüft?<br><br>**Erfüllt? ☐**
7.8.4	Ein System für die Bestandskontrolle von Reagenzien/Verbrauchsgüter existiert. Nicht verwendbare Reagenzien/Verbrauchsgüter werden von für Einsatz akzeptierte getrennt.<br><br>Öffnungs- und Verfallsdatum werden auf dem Behälter vermerkt. Bei Zellkulturen werden Identitätsprüfungen regelmäßig durchgeführt.	Übersicht über Qualität der Reagenzien im Labor. Bei Veröffentlichungen ist es einfacher, Daten heranzuziehen.<br>Ordnungsgemäße Lagerung sicherstellen. Verhinderung von Nutzen von nicht funktionsfähigen Verbrauchsgütern.	Verfahren für Bestandskontrolle einführen. Öffnungs- und Verfallsdatum sind auf dem Behälter vermerkt. Nicht mehr verwendbare Reagenzien werden entsorgt oder auf Nutzbarkeit (Bspw.: Identität, Reinheit, Gehalt) geprüft.<br><br>Gibt es ein Verfahren für die **Bestandskontrolle**?<br>Werden **Öffnungs- und Verfallsdatum** überwacht?<br>Werden **Zellkulturen** auf Identität geprüft?<br><br>**Erfüllt? ☐**
7.8.5	Gebrauchsanweisungen sind leicht zugänglich (einschl. der Herstellergebrauchsanweisungen).	Im Zweifel kann man sich hier informieren. Ordnungsgemäß Anwendung sicherstellen.	Gebrauchsanweisungen sind aufzuheben.<br><br>Sind die **Gebrauchsanweisungen für Reagenzien und Verbrauchsmaterial** leicht zugänglich?<br><br>**Erfüllt? ☐**

7.8.6	Vorkommnisse mit Reagenzien / Verbrauchsgütern werden dem Hersteller / der Behörde gemeldet.	Allgemeine gesetzliche Anforderung.	Vorkommnisse mit Reagenzien und Verbrauchsgütern melden. Verfahren hierfür einführen und Zuständigkeiten klären.<br><br>**Vorgehen für Dokumentation:**<br>• Zuständigkeit für die Meldung von Vorkommnissen an die Behörden klären.<br><br>Werden **Vorkommnisse mit Reagenzien und Verbrauchsgütern gemeldet** (Behörde, Hersteller)?<br>Sind die Zuständigkeiten definiert?<br><br>**Erfüllt? ☐**
7.8.7	Aufzeichnungen über jedes Reagenz / Verbrauchsgut werden geführt, die zur Leistungsfähigkeit der Untersuchungsverfahren beitragen. Inhalt:<br>a) Identitätsbezeichnung des Reagenz oder der Verbrauchsgüter;<br>b) Name des Herstellers und Chargencode oder Losnummer;<br>c) Kontaktangaben des Lieferanten oder des Herstellers;<br>d) Datum der Lieferung, Ablaufdatum, Datum der Inbetriebnahme und falls anwendbar das Datum der Außerbetriebnahme;<br>e) Zustand bei Erhalt (z. B. annehmbar oder beschädigt);<br>f) Anweisungen des Herstellers;<br>g) Aufzeichnungen, die die anfängliche Gebrauchstauglichkeit des Reagenz oder der Verbrauchsgüter bestätigen;<br>h) Aufzeichnungen über die Leistungsfähigkeit, die die andauernde	Aufzeichnungen dienen der Übersicht und Kontrolle über das Lager. Für Publikationen sind die Daten schnell zugänglich.	**Vorgehen für Dokumentation:**<br>• Aufzeichnungen über Verbrauchsmaterialien führen<br><br>Werden entsprechende **Aufzeichnungen über Reagenzien/Verbrauchsgüter** geführt (bei Inhouse Herstellungen von Reagenzien zusätzlich auch Herstellende und Datum der Herstellung notieren)?<br><br>**Erfüllt? ☐**

Gebrauchstauglichkeit des Reagenz oder der Verbrauchsgüter bestätigen.<br><br>Bei Nutzung von im Haus hergestellten oder fertig gestellten Reagenzien sind zusätzlich zu den oben genannten relevanten Angaben Aufzeichnungen mit Hinweis auf die Person und das Datum enthalten, die die Herstellung vornimmt.		

7.9 Extern bereitgestellte Produkte und Dienstleistungen

Forderung	Ziel der Forderung	Umsetzung
Ein Verfahren für die Auswahl von extern bereitgestellten Produkten und Dienstleistern ist etabliert. Das Verfahren stellt sicher, dass folgende Bedingungen erfüllt werden:<br>a) Anforderungen des Laboratoriums für extern bereitgestellte Produkte und Dienstleistungen sind erfüllt<br>b) Das Laboratorium ist verantwortlich für:<br>a. Auswahl der Dienstleister;<br>b. Überwachung der Qualität der Leistung;<br>c. Sicherstellung, dass die Dienstleister die erforderliche Kompetenz besitzen.<br>c) Vereinbarungen mit Dienstleistern werden regelmäßig überprüft und bewertet.<br>d) Es wird ein Verzeichnis aller beauftragten Dienstleister unterhalten.	Gute und schlechte Lieferanten identifizieren. Schlechte Lieferanten können soweit möglich zukünftig gemieden werden.<br><br>Den Ergebnissen von beauftragten dritten Laboratorien / Beratern muss man Vertrauen geschenkt werden können.	Verfahren für die Beschaffung einführen. Lieferanten bewerten.<br><br>**Vorgehen für Dokumentation:**<br>• Prozess für Beschaffung etablieren und dokumentieren<br>• Lieferantenübersicht und Bewertung führen<br><br>Gibt es ein dokumentiertes Verfahren für die **Beschaffung**?<br>Gibt es ein Verfahren für **Auswahl/Bewertung** von Auftragslaboren/ Beratern und ein zugehöriges Verzeichnis?<br>Wird eine **Lieferantenübersicht** geführt (Bewertung, Vorkommnisse)?<br><br>**Erfüllt?** ☐

7.10 Informationsmanagement des Laboratoriums

	Forderung	Ziel der Forderung	Umsetzung
7.10.1	Computergestützte und nicht computergestützte Systeminformationsmanagementsysteme (Erfassung, Verarbeitung, Aufzeichnung, Berichten, Lagerung oder Abfrage von Daten) werden vor Einführung auf Funktionsfähigkeit überprüft werden.	Funktion und Sicherheit von IT-Anwendungen sicherstellen.	Zumeist durch die Organisation gewährleistet.
7.10.2	Das Informationsmanagementsystem ist:<br>a) vor unbefugtem Zugriff geschützt;<br>b) gegen Manipulation/Verlust gesichert;<br>c) so aufrechterhalten, dass Unversehrtheit der Daten sichergestellt wird;<br>d) Aufzeichnungen von Systemausfällen (Sofort- und Korrekturmaßnahmen);<br>e) in Übereinstimmung mit nationalen oder internationalen Anforderungen bezüglich des Datenschutzes sein.		
7.10.3	Anweisungen, Handbücher und Referenzdaten zum Informationsmanagementsystem sind dem Personal leicht zugänglich.		**Erfüllt? ☐**

8 Forschungsprojekt

8.1 Planung eines Forschungsprojekts

8.1.1 Allgemeines

	Forderung	Ziel der Forderung	Umsetzung
8.1.1.1	Bei der Planung des Forschungsvorhabens ist der aktuelle Forschungsstand umfassend zu berücksichtigen und anzuerkennen (GWP 9).	Einhaltung der GWP bei der Planung von Forschungsvorhaben. Die Ressourcen für das Forschungsvorhaben müssen vorhanden sein. Das Personal muss für die geplanten	Einhaltung der GWP-Forderungen.
8.1.1.2	Eine Identifikation relevanter und geeigneter Forschungsfragen setzt eine sorgfältige		

	Recherche nach bereits öffentlich zugänglich gemachten Forschungsleistungen voraus. (GWP 9)	Versuche kompetent sein. Gesetze sollen eingehalten und Genehmigungen eingeholt werden (Bspw.: Auflagen des Ethikrates befolgen).	Wird **die GWP** bei der **Planung** eines Forschungsvorhabens eingehalten? (Recherche aktueller Forschungsstand)<br>Sind alle **Planungsunterlagen** und **Genehmigungen/Anträge** pro Forschungsprojekt an einem Ort?<br>Hat das Personal alle benötigten **Schulungen**, fehlen **Kompetenzen** für das Forschungsprojekt?
8.1.1.3	Rechte und Pflichten, insbesondere solche, die aus gesetzlichen Vorgaben oder auch Verträgen mit Dritten resultieren, sind zu beachten. Dazu zählen auch, soweit erforderlich, die Einholung und Vorlage von Genehmigungen und Ethikvoten (GWP 10).		
8.1.1.4	Das Laboratorium muss über die Leistungsfähigkeit und die Ressourcen verfügen, um das Forschungsprojekt durchzuführen.		**Erfüllt? ☐**
8.1.1.5	Das Personal des Laboratoriums muss über die zur Durchführung der vorgesehenen Untersuchungen erforderlichen Fertigkeiten und Erfahrungen verfügen.		

8.1.2 Planung und Versuchsdesign

	Forderung	Ziel der Forderung	Umsetzung
8.1.2.1	Das Forschungsprojekt und die Fragestellung sind klar zu benennen.	Vor Beginn der Versuche sollten die organisatorischen Fragestellungen des Forschungsprojekts durchdacht werden. Dadurch wird das Risiko des Forschungsprojekts gesenkt. Das spart Zeit, Ressourcen und führt schneller zu einem Ergebnis/ einer Publikation (schließt Abzweigungen des Projekts nicht aus).<br><br>An die Qualitätssicherung und Statistik sollte bereits in der Planungsphase gedacht werden. Sind die Voraussetzungen für die statistische	**Vorgehen für Dokumentation:**<br>• Klare Benennung Forschungsprojekt und Fragestellung<br>• Versuchsplan<br>• Ressourcenplanung<br>• Datenmanagementplan<br>• Überlegungen zur Statistik, Methoden zur Vermeidung von (unbewussten) Verzerrungen<br>• Dual Use<br>• Sind Referenzbereiche festzulegen?<br>• Sind die Nutzungsrechte von generierten Daten und Ergebnissen geklärt und dokumentiert?
8.1.2.2	Es sind grundlegende Parameter der Forschungsarbeit zu definieren:<br>a) Versuchsplanung;<br>b) Ressourcenplanung;<br>c) Datenmanagementplan.		
8.1.2.3	Die Dual Use des Forschungsprojektes ist zu bewerten. (GWP 10)		
8.1.2.4	Soweit möglich, sind bei der Planung Methoden zur Vermeidung von (unbewussten) Verzerrungen bei der Interpretation von Ergebnissen anzuwenden (z. B. Verblindung von Versuchsreihen). (GWP 9)		

8.1.2.5	Die Wahl des Modellsystems beruht auf gründlichen Überlegungen über die Vorteile und Einschränkungen des Modells. Die verwendeten Modelle und Methoden sind hinreichend etabliert. Vorhandene Standards finden Berücksichtigung (DFG).	Versuchsplanung bereits gegeben? Sind Pilotversuche erst zu machen?<br><br>Entscheidungsregeln und Referenzbereiche sind notwendig, um die Aussagekraft der Publikation zu unterstützen (Daten und Ergebnisse sind nicht „an den Haaren herbeigezogen“ und nicht übertrieben dargestellt, sondern belegbar). Durch Dokumentation der verwendeten Entscheidungsregeln gibt es eine Sicherstellung der Nachvollziehbarkeit.	
8.1.2.6	Vor Beginn einer Untersuchung werden Überlegungen zur Statistik geführt (Bspw.: Sind die Voraussetzungen für die statistische Versuchsplanung bereits gegeben? Sind Pilotversuche vorzuziehen? Fallzahlplanung bzw. eine Stichprobenumfangsplanung und Mehrfachkorrektur) (DFG).		
8.1.2.7	Entscheidungsregeln oder Referenzbereiche sind eindeutig definiert und dokumentiert. Deren Grundlage ist auch aufgezeichnet.<br><br>Sollten sie nicht länger aktuell sein, werden Veränderungen nachvollziehbar vorgenommen (Datum und Person). Änderungen der Referenzbereiche werden kommuniziert.		**Erfüllt? ☐**
8.1.2.8	Es werden die Vereinbarungen über Nutzungsrechte der aus ihm hervorgehenden Forschungsdaten und -ergebnissen dokumentiert. (GWP 10)		

8.1.3 Auswahl der Methoden

	Forderung	Ziel der Forderung	Umsetzung
8.1.3.1	Es sind fundierte und wissenschaftliche Methoden anzuwenden. (GWP 11)	Nachvollziehbare Dokumentation der angewendeten Methodik. Durch Arbeitsanweisungen, sind Methoden leichter publizierbar und der Mitarbeiter bzw. der Leiter weiß, wie die Methode auszuführen	• Fundierte und wissenschaftliche Methoden anwenden<br>• Validierte Methoden verwenden<br>• Auf Qualitätssicherung achten<br>• Nachvollziehbarkeit und dadurch Replizierbarkeit gewährleisten
8.1.3.2	Bei der Entwicklung neuer Methoden ist besonderer Wert auf die Qualitätssicherung und die Etablierung von Standards zu legen. (GWP 11)		

8.1.3.3	Untersuchungsverfahren und Begleitdokumentation sind so dokumentiert, dass sie auch für Dritte nachvollzogen und repliziert werden können.	ist / ausgeführt wurde (Fehler werden minimiert und Ressourcen geschont). Jedoch sind Arbeitsanweisungen in der Forschung nicht immer sinnvoll. Wenn keine Arbeitsanweisungen vorhanden sind, muss die Methodik nachvollziehbar während des Versuchs dokumentiert werden. Ziel ist, dass später nachvollzogen werden kann, was gemacht wurde.<br>Validierte Methoden benutzen, da die Untersuchungen auch wirklich das Richtige richtig messen sollen.	**Erfüllt?** ☐

8.1.4 Verifizierung

	Forderung	Ziel der Forderung	Umsetzung
8.1.4.1	Bereits bekannte validierte Untersuchungsverfahren werden vor Einführung im Labor in dem Umfang verifiziert, dass die geforderte Leistungsfähigkeit sichergestellt wird.<br>Verifizierungen werden wiederholt, wenn das Verfahren von der herausgebenden Stelle überarbeitet wird.	Verifizierung von Methoden, um sicher zu gehen, dass bereits validierte Methoden in dem eigenen Labor auch funktionieren.	Verifizierung von Methoden.<br><br>**Vorgehen für Dokumentation:**<br>Dokumentation von Verifizierungen<br><br>Sind die Methoden, die benutzt werden sollen **verifiziert oder validiert**?<br>Sind die **Verifizierungen** nachvollziehbar **dokumentiert**?<br><br>**Erfüllt?** ☐
8.1.4.2	Durchführung und Ergebnisse der Verifizierung werden dokumentiert.		

8.1.5 Validierung

	Forderung	Ziel der Forderung	Umsetzung
8.1.5.1	Untersuchungsverfahren der folgenden Quellen werden validiert:<br>a) nicht genormte Verfahren;<br>b) für das Laboratorium gestaltete oder entwickelte Verfahren;<br>c) Standardverfahren, die außerhalb ihres vorgesehenen Anwendungsbereichs benutzt werden;<br>d) validierte und anschließend modifizierte Verfahren.	Neue Methoden validieren. Das ist notwendig um aussagekräftige und verlässliche Daten zu erhalten.<br><br>Es darf von Messverfahren auch abgewichen werden, dann ist alles ordentlich und nachvollziehbar zu dokumentieren, was gemacht wurde (Hinweis auf keine vorhandene Validierung sollte vorhanden sein).	Neue Methoden validieren.<br><br>**Vorgehen für Dokumentation:**<br>Dokumentation von Validierung
8.1.5.2	Die Validierung wird dokumentiert.<br>Folgende Validierungsnachweise werden aufbewahrt:<br>a) angewandte Validierungsverfahren;<br>b) Bestimmung Leistungsmerkmale;<br>c) erhaltenen Ergebnisse.		Sind die Methoden, die benutzt werden sollen **validiert**?<br>Wird die **Validierung dokumentiert**?<br>Werden bei Änderungen von validierten Verfahren, die Einflüsse der Änderung dokumentiert oder ggf. erneut validiert?
8.1.5.3	Bei Änderung an validierten Verfahren wird der Einfluss der Änderung dokumentiert und ggf. neu validiert.		**Erfüllt?** ☐
8.1.5.4	Die überprüften Leistungsmerkmale (z.B. Genauigkeit, Nachweisgrenze, Linearität, Robustheit, etc.) sind dem Projekt angemessen.		

8.1.6 Messunsicherheit von gemessenen Größenwerten

	Forderung	Ziel der Forderung	Umsetzung
8.1.6.1	Messunsicherheiten von Untersuchungsverfahren werden ermittelt.	Messunsicherheiten bei der Auswertung beachten.	Messunsicherheiten berücksichtigen.<br><br>Werden **Messunsicherheiten** von Untersuchungsverfahren ermittelt und in der Interpretation beachtet?
8.1.6.2	Bei der Interpretation der Ergebnisse wird die Messunsicherheit berücksichtigt.		
8.1.6.3	Bei Untersuchungen, die einen Messschritt enthalten, aber keinen Größenwert ausgeben, sollte die Messunsicherheit berechnet		**Erfüllt?** ☐

	werden (wenn es für Beurteilung der Zuverlässigkeit des Untersuchungsverfahrens sinnvoll ist).		

8.2 Durchführung

8.2.1 Allgemeines

	Forderung	Ziel der Forderung	Umsetzung
	Während der Durchführung des Forschungsprojekts wird auf Kennzeichnung (Beschriftung Proben, etc.) und Rückverfolgbarkeit (Dokumentation) geachtet.	Replizierbarkeit und Fehleranalysen auch Jahre später möglich machen. Aussagefähigkeit gegenüber Fragen zur Publikation.<br><br>Kennzeichnung: Verwechslung ausschließen, Erkennung des Bearbeitungsstandes der Probe<br>Rückverfolgbarkeit: später Fehlerursachen und Auswirkungen von Fehlern nachvollziehen können (Publikationen zurückhalten?)	Auf Kennzeichnung (Beschriftung Proben, etc.) und Rückverfolgbarkeit (Dokumentation, sodass auch andere es nachvollziehen können) achten.<br><br>**Erfüllt? ☐**

8.2.2 Dokumentation der Untersuchungsverfahren

	Forderung	Ziel der Forderung	Umsetzung
8.2.2.1	Untersuchungsverfahren werden dokumentiert. Die Dokumentation erfolgt in der Sprache, die für das Personal verständlich ist.	Den Mitarbeitern ist durch Arbeitsanweisungen der Ablauf der Methode / des Prozesses bekannt und kann bei Unklarheiten nachgelesen werden (insbesondere, wenn eine Nachfrage nicht möglich ist (Krankheit, Abwesenheit, etc.)). Das Vorgehen ist im Nachhinein leichter	**Vorgehen für Dokumentation:**<br>Erstellung von Arbeitsanweisungen, wo angebracht. In einem „Dokumentenlogbuch“ Übersicht über Dokumente führen, einschließlich dem Verteilungsort (dann wird beim Austausch keine vergessen).
8.2.2.2	Arbeitsanweisungen existieren dort, wo eine Wissenssicherung notwendig ist und standardisierte Methoden existieren. Wenn für Untersuchungsverfahren keine Arbeitsanweisungen existieren, ist die genaue		

	Durchführung des Verfahrens rückverfolgbar zu dokumentieren.	publizierbar und für die Zukunft archivierbar (Wissenssicherung). Der Projektleiter weiß, wie die Mitarbeiter vorgegangen sind.	Sind die Untersuchungsverfahren als **Arbeitsanweisung dokumentiert**?<br>Wo es nicht sinnvoll ist, Arbeitsanweisungen zu erstellen, werden die durchgeführten Versuche **nachvollziehbar im Laborbuch / in den Aufzeichnungen beschrieben**?<br>Sind Arbeitsanweisungen für Untersuchungsverfahren **zugänglich** für das Personal?<br>Erfolgt eine **Lenkung** der Arbeitsanweisungen?
8.2.2.3	Die Arbeitsanweisungen müssen an entsprechender Stelle zur Verfügung stehen.		
8.2.2.4	Arbeitsanweisungen werden gelenkt.		
8.2.2.5	Arbeitsanweisungen sollten zusätzlich zu den Identifikationsangaben für die Dokumentenlenkung folgenden Inhalt haben:<br>a) Grundlage und Methode des für die Untersuchungen angewendeten Verfahrens;<br>b) Leistungsmerkmale;<br>c) Art der Probe (z. B. Plasma, Serum, Urin);<br>d) Art des Behälters und der Zusatzstoffe;<br>e) erforderliche Ausrüstung und Reagenzien;<br>f) Umwelt- und Sicherheitsmaßnahmen;<br>g) Kalibrierverfahren;<br>h) Schritte im Arbeitsablauf;<br>i) Verfahren der Qualitätssicherung;<br>j) Störungen und Kreuzreaktionen;<br>k) Kurzbeschreibung des Verfahrens zur Ergebnisberechnung einschließlich der Messunsicherheit der gemessenen Größenwerte, falls zutreffend;<br>l) Referenzbereiche oder Entscheidungswerte;<br>m) mögliche Ursachen von Abweichungen;<br>n) Verweise.	Arbeitsanweisungen müssen im Umfang den Anforderungen des Labors genügen.	**Erfüllt? ☐**

8.2.3 Probenahme

	Forderung	Ziel der Forderung	Umsetzung
8.2.3.1	Soweit möglich, sind bei der Probenahme Methoden zur Vermeidung von (unbewussten) Verzerrungen bei der Interpretation von	Sorgfältige Probenahme um Qualität sicherzustellen und	**Vorgehen für Dokumentation:**<br>Probenahme dokumentieren und Anweisungen für die Abnahme unterschiedlicher Proben verfassen.

	Ergebnissen anzuwenden (z. B. Verblindung von Versuchsreihen). (GWP 9)	Ressourcenverschwendung zu reduzieren (Kosten, Zeit, Material).<br>Schon bei der Probenahme Methoden zur Vermeidung von (unbewussten) Verzerrungen der Ergebnisse anwenden.
8.2.3.2	Die Probenahmepläne beruhen, sofern anwendbar, auf angemessenen statistischen Modellen.	
8.2.3.3	Es gibt ein dokumentiertes Verfahren für eine ordnungsgemäße Probennahme. Das Verfahren stellt die Validität der Ergebnisse sicher.	
8.2.3.4	Inhalt von Arbeitsanweisungen für Entnahmetätigkeit:<br>a) Art der Probe;<br>b) Behälter für die Aufnahme von Primärproben und aller erforderlichen Zusatzstoffe;<br>c) Kennzeichnung von Primärproben<br>d) Aufzeichnung der Identität der die Primärprobe entnehmenden Person und das Entnahmedatum und Entnahmezeitpunkts;<br>e) sachgerechte Lagerungsbedingungen, bevor die entnommenen Proben an das Laboratorium versandt werden;<br>f) sichere Entsorgung der bei der Entnahme.	
8.2.3.5	Die Dokumentation der Probennahme stellt eine Rückverfolgbarkeit sicher.<br>Aufzeichnungen der Daten zur Probenahme werden aufbewahrt. Die Aufzeichnungen enthalten folgendes, wo passend:<br>a) angewendete Verfahren;<br>b) Datum und Uhrzeit der Probenahme;<br>c) Identifizierung und Beschreibung der Probe (z.B. Nummer, Menge, Beschreibung);<br>d) Personal, das die Probe nimmt;	

e) verwendete Materialien;<br>f) Umgebungsbedingungen, Transportbedingungen;<br>g) Ort der Probennahme (wo am Körper, im Boden, an welcher Stelle in welchem Gewässer, …);<br>h) Abweichungen, Ergänzungen, Ausschlüsse von Probenahmeverfahren und Plan.		Gibt es ein **dokumentiertes Verfahren** für ordnungsgemäße **Probennahme**, Probenahmepläne (Validität der Ergebnisse sicherstellen, Statistik beachten)?<br>Gibt es **Aufzeichnungen** über Probenahmen?<br><br>**Erfüllt?** ☐

8.2.4 Aufzeichnungen

	Forderung	Ziel der Forderung	Umsetzung
8.2.4.1	Wissenschaftler dokumentieren alle für das Zustandekommen eines Forschungsergebnisses relevanten Informationen so nachvollziehbar, wie dies im betroffenen Fachgebiet erforderlich und angemessen ist, um das Ergebnis überprüfen und bewerten zu können. (GWP 12).	Rückverfolgbare und nachvollziehbare Aufzeichnungen stellen eine Grundanforderung an Forschende dar. Sie machen die Auswertung und Publikation einfacher. Auskunftsfähigkeit für eventuelle Nachfragen durch dritte Wissenschaftler. Spätere Fehler- und Ursachensuche ermöglichen.	• Nachvollziehbare Dokumentation des Vorgehens, Reproduzierbarkeit und Rückverfolgbarkeit (einschließlich Datum und Identität der Labormitarbeiter, (Identität für Nachfragen)) sicherstellen<br>• Ablageort und Datenübermittlung von durchführenden Laborpersonal an verantwortlichen Wissenschaftler festlegen<br>• Daten vor unbeabsichtigter Änderung schützen<br>• nachvollziehbare und rückverfolgbare Änderungen an technischen Aufzeichnungen<br><br>**Vorgehen für Dokumentation:**<br>Ein elektronisches Laborbuch sollte diese Punkte bereits erfüllen.
8.2.4.2	Grundsätzlich sollen auch Einzelergebnisse dokumentiert werden, die die Forschungshypothese nicht stützen. Eine Selektion von Forschungsergebnissen hat zu unterbleiben. (GWP 12)		
8.2.4.3	Technische Aufzeichnungen lassen jegliche Reproduzierbarkeit und Rückverfolgbarkeit zu, einschließlich Datum und Identität der Labormitarbeiter.		
8.2.4.4	Beobachtungen, Daten und Berechnungen werden zu dem Zeitpunkt, zu dem sie gemacht werden, aufgezeichnet und der speziellen Aufgabe zugeordnet.		
8.2.4.5	Änderungen an technischen Aufzeichnungen zu früheren Versionen oder ursprünglichen		

	Beobachtungen können nachvollzogen werden (wer, wann, was, wo geändert hat). Sowohl die ursprünglichen als auch die geänderten Daten werden aufbewahrt.		**Erfüllt?** ☐
8.2.4.6	Dokumentationen und Forschungsergebnisse dürfen nicht manipuliert werden; sie sind bestmöglich gegen Manipulationen zu schützen. (GWP 12). Elektronische Daten werden vor unbeabsichtigter Änderung geschützt.		

8.2.5 Qualitätssicherungsmaßnahmen

	Forderung	Ziel der Forderung	Umsetzung
8.2.5.1	Ein Verfahren zur Qualitätssicherung der Ergebnisse ist vorhanden.<br>Dieses wird dokumentiert und so aufgezeichnet, dass eventuelle Tendenzen erkennbar sind.	Qualitätssicherung der Ergebnisse mit bedenken und dokumentieren. Validität der Ergebnisse sind durch qualitätssichernde Maßnahmen belegbar. Aussagekraft erhöhen.	• geeignete Maßnahmen zur Qualitätssicherung der Ergebnisse vornehmen und dokumentieren<br>• regelmäßige Prüfung der Qualitätskontrollmaterialien<br>• Verfahren, das bei Versagen der Qualitätskontrolle weitere Maßnahmen einleitet<br>• Qualitätskontrolle der Methodendurchführung durch unabhängige Reproduktion von Schlüsselergebnissen<br><br>**Vorgehen für Dokumentation:**<br>• Qualitätssicherung des Versuchs dokumentieren<br>• Prüfung der Referenzmaterialien dokumentieren
8.2.5.2	Das Labor nimmt geeignete Maßnahmen zur Qualitätssicherung vor:<br>a) Verwendung von Referenzmaterialien oder Materialien zur Qualitätssicherung;<br>b) Nutzung von alternativen Messausrüstungen, die kalibriert werden, um rückführbare Ergebnisse bereitzustellen;<br>c) Funktionsprüfung der Mess- und Prüfeinrichtungen;<br>d) Zwischenprüfungen der Messeinrichtungen;<br>e) Wiederholungsprüfungen oder -kalibrierungen unter Anwendung derselben oder unterschiedlicher Verfahren;<br>f) erneute Prüfung oder Kalibrierung von aufbewahrten Gegenständen;<br>g) Vergleiche innerhalb des Laboratoriums;<br>h) Blindversuch.		

8.2.5.3	Eine regelmäßige Prüfung der Referenz- und Qualitätskontrollmaterialien (Prüfung beruht auf Stabilität des Verfahrens, Risiko) erfolgt.		
8.2.5.4	Das Labor verfügt über ein Verfahren, das bei Versagen der Qualitätskontrolle weitere Maßnahmen einleitet.<br>Die Daten der Qualitätssicherung werden regelmäßig bewertet, um auf Trends in Untersuchungsleistungen reagieren zu können und Maßnahmen zu ergreifen und zu dokumentieren.		**Erfüllt? ☐**

8.3 Auswertung

	Forderung	Ziel der Forderung	Umsetzung
8.3.1	Die Auswertung und Interpretation der Daten werden nachvollziehbar und nach dem Stand der Technik dokumentiert.	Eine logische, nachvollziehbare Dokumentation hilft beim späteren Nachvollziehen des Projekts. Es muss hinterfragt werden, ob die Auswertungssoftware richtig funktioniert.	Überprüfung der eigenen Dokumentationsart und der Auswertungssoftware.<br><br>Sind die Auswertungen/Interpretationen eines Projekts auch für Dritte **nachvollziehbar dokumentiert und abgespeichert**?<br>Sind die **Auswertungsprogramme validiert** (stimmt das Ergebnis, was sie berechnen)?<br><br>**Erfüllt? ☐**
8.3.2	Es werden validierte Auswertungsprogramme verwendet.		

8.4 Publikation

8.4.1 Beschreibung der Methoden und Analysen

	Forderung	Ziel der Forderung	Umsetzung
8.4.1.1	Soweit möglich und zumutbar sind die den Ergebnissen zugrundeliegenden	Die Publikation muss für dritte Wissenschaftler nachvollziehbar sein.	Einhaltung der GWP.

	Forschungsdaten, Materialien und Informationen, die angewandten Methoden sowie die eingesetzte Software verfügbar zu machen und Arbeitsabläufe umfänglich darzulegen. (GWP 13)	Anhand der Publikation sollten Wissenschaftler den Versuch reproduzieren können.<br>Betrug, Übertreiben und Aufblähen der eigenen Forschung leisten keinen Beitrag und fördert die Ressourcenverschwendung.	Sind **Forschungsdaten, Materialien, Informationen, angewandten Methoden**, **eingesetzte Software** sowie die Arbeitsabläufe nachvollziehbar dargelegt?<br>Sind die **Hypothesen** und die **statistische Methodik** präzise dargestellt?<br>Werden **alle Ergebnisse** eingebracht?<br><br>**Erfüllt?** ☐
8.4.1.2	Es erfolgt eine umfassende Darstellung der Methoden und Analysen. Hierzu gehören insbesondere eine sorgfältige, vollständige und genaue Darstellung der Hypothesen und statistischen Methodik der Auswertungen in Berichten und Publikationen. (DFG)		
8.4.1.3	Grundsätzlich bringen Wissenschaftler alle Ergebnisse in den wissenschaftlichen Diskurs ein. Im Einzelfall kann es Gründe geben, Ergebnisse nicht öffentlich zugänglich zu machen; dabei darf diese Entscheidung nicht von Dritten abhängen (GWP 13).		
8.4.1.4	Der selektive Blick auf ausgewählte Ergebnisse sowie das post-hoc Anpassen der Hypothese an Ergebnisse stellen eine massive Beeinträchtigung der Aussagekraft von Ergebnissen dar und hat zu unterbleiben. (DFG)		

8.4.2 Beschreibung Qualitätssicherung

Forderung	Ziel der Forderung	Umsetzung
Wenn wissenschaftliche Erkenntnisse öffentlich zugänglich gemacht werden (z. B. über Publikationen oder andere Kommunikationswege) sind – insbesondere, wenn neue Methoden entwickelt werden - die angewandten Mechanismen der forschungsbegleitenden Qualitätssicherung darzulegen. (GWP 7)	Aussagekraft des Forschungsprojekts durch Beschreibung der forschungsbegleitenden Qualitätssicherung verbessert.	GWP einhalten.<br><br>Wurden angewandte Mechanismen der **forschungsbegleitenden Qualitätssicherung** beschrieben (insbesondere, wenn neue Methoden entwickelt werden)?<br><br>**Erfüllt?** ☐

8.4.3 Replizierbarkeit

	Forderung	Ziel der Forderung	Umsetzung
8.4.3.1	Werden Ergebnisse öffentlich zugänglich gemacht, müssen diese vollständig und nachvollziehbar beschrieben werden. (GWP 13)<br>Die Ergebnisse müssen unabhängig repliziert werden können (z.B. mittels ausführlicher Beschreibungen von Materialien und Methoden) (GWP 7).	Eine Selbstprüfung und /-hinterfragung der Ergebnisse für die Publikation kann bereits Unstimmigkeiten aufdecken.	Die Publikation selbstständig auf Nachvollziehbarkeit prüfen.<br>**Erfüllt?** ☐
8.4.3.2	Die Publikation wird vor Herausgabe auf Replizierbarkeit und Nachvollziehbarkeit geprüft.		

8.4.4 Nachweis von Vorarbeiten

	Forderung	Ziel der Forderung	Umsetzung
	Eigene und fremde Vorarbeiten werden vollständig und korrekt nachgewiesen. (GWP 13)	Plagiat und damit Betrug und Reputationsschaden verhindern.<br>Replikation von Versuchen angeben, um wissenschaftliche Erkenntnisse der wissenschaftlichen Gemeinschaft zu festigen oder zu hinterfragen.	Eigene und fremde Vorarbeiten vollständig und korrekt nachweisen.<br>**Erfüllt?** ☐

8.4.5 Zustimmung der Autoren

	Forderung	Ziel der Forderung	Umsetzung
8.4.5.1	Die Autoren haben einen wesentlichen wissenschaftlichen Beitrag zur Veröffentlichung geleistet (GWP 14).	Konflikte im Vorhinein verhindern.	Alle Autoren die Publikation prüfen lassen und Zustimmung einholen.<br><br>**Vorgehen für Dokumentation**:<br>• Zustimmungen mit Projekt archivieren
8.4.5.2	Es hat eine Zustimmung aller Autoren zu der finalen Fassung zu erfolgen (GWP 14).		
8.4.5.3	Allen Autoren wurde die Möglichkeit zur Prüfung der Veröffentlichung gegeben.		

Haben alle **Autoren** zur Veröffentlichung **zugestimmt** und hatten sie vorher die Möglichkeit zur Prüfung der Veröffentlichung (Zustimmung dokumentieren, z.B. E-Mail-Verlauf dokumentieren)?

Erfüllt? ☐

8.4.6 Publikationsorgan

	Forderung	Ziel der Forderung	Umsetzung
8.4.6.1	Autoren wählen das Publikationsorgan – unter Berücksichtigung seiner Qualität und Sichtbarkeit im jeweiligen Diskursfeld – sorgfältig aus (GWP 15).<br>Sogenannte „Predatory Journals" sind zur Veröffentlichung von Forschungsergebnissen zu vermeiden.	Erhöhung der Reichweite der Publikation durch Prüfung von Seriosität und Leserkreis.	„Predatory Journals" sind zur Veröffentlichung von Forschungsergebnissen zu vermeiden. Neue oder unbekannte Publikationsorgane auf Seriosität prüfen.<br><br>**Erfüllt? ☐**
8.4.6.2	Es besteht eine Pflicht, neue oder unbekannte Publikationsorgane auf ihre Seriosität hin zu prüfen. (GWP 15)		

8.4.7 Berichtigung veröffentlichter Ergebnisse

	Forderung	Ziel der Forderung	Umsetzung
8.4.7.1	Wenn Wissenschaftlern Unstimmigkeiten oder Fehler zu von Ihnen veröffentlichten Ergebnissen bekannt werden, berichtigen sie diese. (GWP 7)	Publizierte Fehler werden berichtigt und nicht verschwiegen oder unbeachtet gelassen. Seriosität der eigenen Forschung sicherstellen.<br><br>Aus Fehlern soll gelernt werden. Hierfür wird nach der Ursache gesucht und entsprechende Maßnahmen eingeleitet, damit in Zukunft eine Wiederholung verhindert wird.	Wenn Fehler einer Publikation bekannt werden, werden diese durch das Abweichungsmanagement bearbeitet.<br><br>**Vorgehen für Dokumentation:**<br>• Dokumentation und Bearbeitung im Abweichungsmanagement (s. 9.3)<br><br>Werden bei **Unstimmigkeiten oder Fehlern von veröffentlichten Ergebnissen**, diese berichtigt (Ursache des Fehlers analysieren und Korrekturmaßnahmen einleiten)?<br><br>**Erfüllt? ☐**
8.4.7.2	Die Ursache des Fehlers wird analysiert und entsprechende Korrekturmaßnamen ergriffen.		

8.5 Archivierung

	Forderung	Ziel der Forderung	Umsetzung
8.5.1	Wissenschaftler sichern Forschungsdaten und -ergebnisse sowie die ihnen zugrunde liegenden zentralen Materialien in adäquater Weise und bewahren sie gemäß gesetzlichen Vorgaben und für einen angemessenen Zeitraum (in der Regel 10 Jahre) zugänglich und nachvollziehbar auf. (GWP 17). Ggf. verkürzte Aufbewahrungszeiten sind plausibel zu begründen (GWP 17).	Publizierte Daten sollten auch in späterer Zukunft immer bewiesen und nachvollzogen werden können. Der Forscher soll für Fragen und Antworten auskunftsfähig sein. Das ist für Reputation und Seriosität wichtig.	Nachvollziehbare Archivierung von Daten und Material gemäß gesetzlichen Vorgaben (mind. 10 Jahre).<br><br>Sind Forschungsprojekte gemäß **gesetzlichen Vorgaben und mind. 10 Jahre nachvollziehbar** archiviert?<br>Gibt es dokumentierte Begründung für **verkürzte Aufbewahrungszeiten**?<br>Sind verwendete Materialien und entstehende Forschungsdaten so aufbewahrt, dass sie für andere **zur Verfügung gestellt** werden könnten?<br>Ist der **Zugriff** auf das Archiv eingeschränkt?<br><br>**Erfüllt? ☐**
8.5.2	Die verwendeten Materialien und entstehenden Forschungsdaten werden in geeigneten Daten- und Gewebebanken zur Wiederholung, Überprüfung und Nachnutzbarkeit für andere zur Verfügung gestellt (DFG).		
8.5.3	Sämtliche elektronische Daten einschließlich Korrespondenzen werden gesichert. Die Datenablage hat eine logische und nachvollziehbare Struktur.		

9 Anforderungen an das Managementsystem

9.1 Lenkung von Dokumenten des Managementsystems

	Forderung	Ziel der Forderung	Umsetzung
9.1.1	Vorgabedokumente werden gelenkt.	Da zufällige Entdeckungen in der Forschung durch „Fehler“ oder ausprobieren nicht verhindert werden sollen, dienen die Arbeitsanweisungen in der Forschung der Beschreibung des allgemeinen Ablaufs (insb. für Personen, die das Verfahren	• Lenkung von Vorgabedokumenten, Prozessbeschreibungen etc.<br>• aktuelle Dokumente anwenden → falls das nicht anwendbar, verwendete Version und / oder allen Abweichungen von der aktuellen Version notieren<br>• Dokumente durch Ersteller und Prüfer freigeben
9.1.2	Es werden nur aktuelle Dokumente angewendet. Falls Abweichungen von dem Vorgabedokument notwendig sind, werden die verwendete Version und alle Abweichungen von der aktuellen Version notiert.		

9.1.3	Neue Dokumente werden von dem Ersteller und mindestens einem weiteren Prüfer freigegeben. Das Personal ist hierfür kompetent und befugt.
9.1.4	Dokumente werden eindeutig benannt.
9.1.5	Relevante Versionen von anwendbaren Dokumenten sind dort verfügbar, wo sie genutzt werden.
9.1.6	Änderungen und der aktuelle Revisionsstatus der Dokumente können identifiziert werden. Handschriftliche Änderungen von Dokumenten werden mit Datum und Person versehen. Diese werden in eine neue Version überschrieben.
9.1.7	Dokumente werden regelmäßig überprüft und in einem Abstand zu aktualisieren, der sicherstellt, dass sie für die „Verwendung geeignet" bleiben.
9.1.8	Mindestens eine Kopie eines veralteten gelenkten Dokuments wird für einen bestimmten Zeitraum oder mindestens der Archivierungsdauer des Projektes aufbewahrt.

noch nicht oft durchgeführt haben). Des Weiteren können anhand von Arbeitsanweisungen Arbeitsschritte der Vergangenheit besser nachvollzogen werden. Wichtig ist, dass abweichende Durchführungen der Methode gut und nachvollziehbar dokumentiert werden, aber keinesfalls verboten sind.
Material und Methoden in Publikationen schneller beschreibbar.
Durch Arbeitsanweisungen auch in Zukunft nachvollziehbar, wie eine Methode durchgeführt wurde.

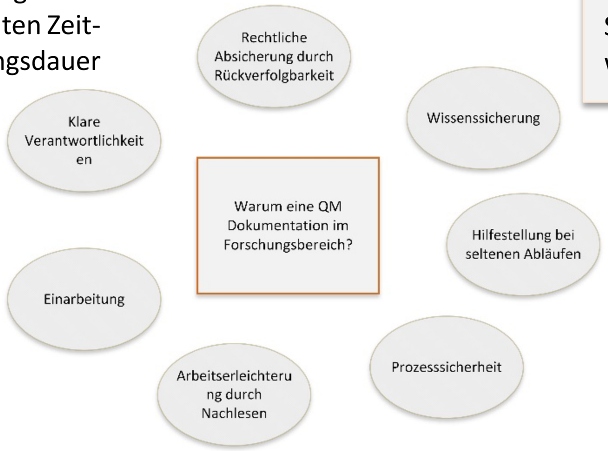

Abbildung 5 Vorteile QM-Dokumentation in der Forschung

- Dokumente eindeutig benennen (ID-Code)
- Dokumente dort verfügbar machen, wo sie genutzt werden
- Änderungen an Dokumenten zu Vorversionen sind sichtbar
- Dokumente regelmäßig auf Aktualität prüfen und wenn nötig überarbeiten
- Eine Kopie des veralteten Dokuments archivieren

Vorgehen für Dokumentation:

- Verfassen von Verfahrensanweisungen für Dokumentenlenkung
- Verfassen einer allgemeinen Dokumentenübersicht

Werden **aktuelle Vorgabedokumente** angewendet und gelenkt?
Werden diese durch ein **Mehraugenprinzip** geprüft?
Sind Vorgabedokumente **eindeutig benannt**?
Sind Vorgabedokumente dort **verfügbar**, wo sie genutzt werden?
Sind **Änderungen** zur Vorversion **erkennbar**?
Wird die Vorversion **archiviert**?

Erfüllt? ☐

9.2 Lenkung von Aufzeichnungen

	Forderung	Ziel der Forderung	Umsetzung
9.2.1	Es sind Maßnahmen eingeführt, die für:<br>a) Kennzeichnung;<br>b) Aufbewahrung;<br>c) Schutz;<br>d) Archivierung;<br>e) Wiederauffindbarkeit;<br>f) Aufbewahrungsfrist;<br>g) Vernichtung<br>der Aufzeichnungen erforderlich sind.	Aufzeichnungen eines Projekts sind schnell auffindbar, bei übersichtlicher Ablage sind Daten schneller auswertbar, der Forschungsprozess ist rückverfolgbar, Schutz vor Datenverlust und nachträglicher Manipulation.	• Aufzeichnung während Versuchs machen<br>• Maßnahmen existent für:<br>• Kennzeichnung<br>• Aufbewahrung<br>• Schutz<br>• Archivierung<br>• Wiederauffindbarkeit<br>• Aufbewahrungsfrist<br>• Vernichtung<br>• Änderung Aufzeichnung: Datum und Person<br><br>Werden **Aufzeichnungen gelenkt**?<br><br>**Erfüllt? ☐**
9.2.2	Für alle Aktivitäten, die die Qualität der Forschung beeinflussen:<br>a) Aufzeichnungen während Verfahrens machen;<br>b) Änderungen von Aufzeichnungen: Datum und Person erfassen.		
9.2.3	Der Zugang zu Aufzeichnungen muss mit Vertraulichkeitsvereinbarungen in Einklang stehen.<br>Aufzeichnungen sind für berechtigte Personen leicht verfügbar.		

9.3 Nichtkonformitäten und Korrekturmaßnahmen

	Forderung	Ziel der Forderung	Umsetzung
9.3.1	Das Labor verfügt über ein Verfahren, das den Umgang mit eigenen Nichtkonformitäten klärt.	Die Forschung ist charakterisiert durch Nichtkonformitäten. Diese können manchmal entscheidend sein (Bspw. Entdeckung Penicillin). Deswegen müssen Nichtkonformitäten in der Forschung anders als in	• Verfahren für Umgang mit Nichtkonformitäten / Fehlern existieren:<br>• Verantwortlichkeiten und Befugnisse definieren<br>• Sofortmaßnahmen umsetzen<br>• Ausmaß des Fehlers feststellen und Maßnahmen einleiten (z.B. Publikation nicht veröffentlichen)
9.3.2	Das Verfahren stellt sicher, dass:<br>a) Verantwortlichkeiten und Befugnisse für Umgang mit Fehlern festgelegt sind;<br>b) Sofortmaßnahmen festgelegt werden;<br>c) das Ausmaß des Fehlers festgestellt wird;		

d) sofern erforderlich, Untersuchungen unterbrochen und Publikationen zurückgehalten werden;

e) erforderlichenfalls die bereits freigegebenen Publikationen fehlerhafter Untersuchungen zurückgerufen oder in geeigneter Weise berichtigt werden;

f) Fehler im Forschungsablauf dokumentiert und aufgezeichnet werden, wobei diese Aufzeichnungen in regelmäßigen Abständen überprüft werden, um Tendenzen aufzudecken und vorbeugende Maßnahmen einzuleiten.

der Diagnostik anders definiert werden.

Eine Möglichkeit zur Definition: „Nichtkonformitäten der Forschung sind Geschehnisse, die den geregelten Forschungsablauf stören.“ Beispiel: Geräteausfall, nochmaliges Durchführen von Versuchen, weil unzureichend aufgezeichnet wurde, falsche Lagerung von Reagenzien und deswegen unbrauchbar, falsche Zellkulturen verwendet, da die eigentlichen überwachsen sind (sehr spät aufgefallen)

- Dokumentation
- Wiederholende Fehler:
  - Ursachenanalyse
  - Korrekturmaßnahmen
  - Wirksamkeit der Korrekturmaßnahmen überprüfen

Knappere Dokumentation als im Diagnostikbereich (z.B. anhand einer einzigen Tabelle und nicht mehreren verschiedenen Formblättern). Es ist wichtig, z.B. Ausfälle von Kühlschränken kurz zu dokumentieren, um nachzuvollziehen wie oft dies tatsächlich vorkommt und ob man hier reagieren muss. Unbewusste systematische Fehler vermeiden.

9.3.3 Wenn sich Fehler wiederholen könnten, werden Maßnahmen zur Feststellung, Dokumentation und Ausschaltung der Ursache ergriffen, Korrekturmaßnahmen festgelegt und dokumentiert.

Die Korrekturmaßnahmen sind dem Fehler angemessen. Ihre Wirksamkeit wird überprüft.

Offene Fehlerkultur leben: Nicht wer ist schuld, sondern was. Das Lernen aus Fehlern führt zu effizienteren Prozessen.

Ziel ist es nicht bewusste systematische Fehler und Störungen des normalen Forschungsablaufs zu vermeiden.

Vorgehen für Dokumentation:
Verfahren für den Umgang und die Bearbeitung von Abweichungen definieren und Abweichungsübersicht und -bearbeitung erstellen

Erfolgt eine **Aufzeichnung von Fehlern**?

Erfüllt? ☐

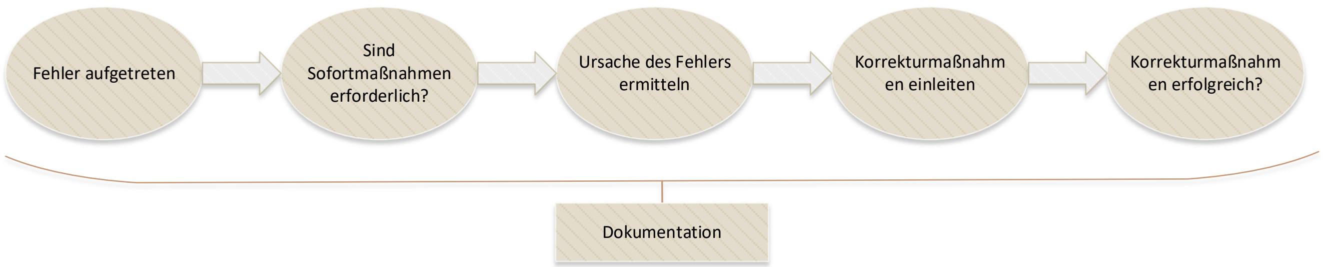

Abbildung 6 Fehler und Korrekturmaßnahmen

9.4 Internes Peer Review

	Forderung	Ziel der Forderung	Umsetzung
9.4.1	In geplanten Abständen werden interne Peer Reviews durchgeführt.	Interne Peer Reviews dienen als kollegialer Austausch zwischen Wissenschaftlern. Hier werden Prozesse durchgesprochen und verbessert und ein Austausch gepflegt (andere Sichtweisen kennen lernen, Horizont erweitern). Mögliche fehlerhafte, doppelt laufende oder aufgeblähte Prozesse werden entdeckt und korrigiert.	• Etablierung und Teilnahme an internen Peer Reviews<br>• Ergebnisse der Leitung mitteilen<br>• geeignete Korrekturen und Korrekturmaßnahmen umsetzen<br>• Dokumentation<br><br>Erfolgt eine Teilnahme an einem **internen Peer Review**?<br><br>**Erfüllt?** ☐
9.4.2	In den internen Peer Reviews wird folgendes geprüft:<br>a) Einhaltung dieses Dokumentes sowie eigener interner Dokumente und Vorgaben;<br>b) Nachvollziehbarkeit von Forschungsprojekten;<br>c) Qualitätsmanagement und -sicherung werden gelebt (Dokumente eingehalten, Konformität sicherstellen).		
9.4.3	Die Auswahl der internen Peer Reviewer und die Durchführung der Peer Reviews stellen eine Objektivität und Unvoreingenommenheit sicher. Es erfolgt ein Austausch auf Augenhöhe.		
9.4.4	Es wird sichergestellt, dass die Ergebnisse des internen Peer Reviews gegenüber der zuständigen Leitung berichtet werden.		
9.4.5	Es werden geeignete Korrekturen und Korrekturmaßnahmen umgesetzt ohne eine Sanktionierung bei Nichtumsetzung.		

9.5 Managementbewertung

	Forderung	Ziel der Forderung	Umsetzung
9.5.1	Die Leitung des Laboratoriums überprüft das Qualitätsmanagementsystem in geplanten Abständen, um dessen fortgesetzte Eignung, Angemessenheit und Wirksamkeit sicherzustellen.	Bin ich mit meinem Team noch auf dem richtigen Weg? Haben wir unsere Ziele erfüllt? Muss etwas angepasst werden?<br><br>Einmal im Jahr schaut sich die Leitung an, was in dem vergangenen Jahr gut und schlecht lief. Daraus	• Laborleitung bewertet in regelmäßigen Abständen (meist ein Jahr) Folgendes und ergreift gegebenenfalls Maßnahmen:<br>• Veränderungen bei externen (Gibt es Veränderungen in Gesetzen, Anforderungen durch Behörden? Gibt es eine neue wissenschaftliche Sensation, die meine Forschung betrifft? Was ist gesellschaftlich gerade interessant?) und internen Angelegenheiten (Gibt es neue Anforderungen,
9.5.2	Die Eingaben in die Managementbewertung enthalten folgende Punkte:		

	a) Veränderungen bei externen und internen Angelegenheiten, die das Forschungslaboratorium betreffen;<br>b) Erfüllung von Zielen;<br>c) Eignung der grundsätzlichen Regelungen und Verfahren;<br>d) Status von Maßnahmen vorheriger Managementbewertungen;<br>e) Ergebnis der jüngsten internen Peer Reviews;<br>f) Korrekturmaßnahmen;<br>g) Begutachtungen von externen Stellen;<br>h) Feedback von Mitarbeitern und Externen;<br>i) Wirksamkeit von jeglichen umgesetzten Verbesserungen;<br>j) Angemessenheit von Ressourcen;<br>k) Ergebnisse der Risikoidentifikation;<br>l) Resultat der Sicherstellung der Validität der Ergebnisse;<br>m) andere relevante Faktoren wie die Überwachungstätigkeiten und Schulungen;<br>n) Leistungsfähigkeit der Lieferanten.	werden Schlüsse gezogen und Maßnahmen eingeleitet, um Prozesse und Abläufe zu optimieren oder gegebenenfalls die Qualitätsziele strategisch neuauszurichten.<br><br>Prozessleistung beurteilen (Lieferantenbewertung, Feedback, Fehler, etc.), QM-System auf Eignung bewerten und anpassen.	die ich erfüllen soll (vielleicht aufgrund Umstrukturierungen<br>• oder Leitungspositionswechsel)), die das Laboratorium betreffen;<br>• Erfüllung von Zielen (Welche Ziele habe ich mir in meiner Forschung gesetzt? Habe ich diese erreicht?)<br>• Eignung der grundsätzlichen Regelungen und Verfahren (Sind die Prozesse in meinem Labor noch in Ordnung?)<br>• Status von Maßnahmen vorheriger Managementbewertungen (Wie war die Bewertung letztes Jahr ausgefallen? Gibt es Veränderungen? Wurden festgelegte Maßnahmen umgesetzt?)<br>• Ergebnis des jüngsten internen Peer Reviews (Wie hat mein Labor im letzten Peer Review abgeschnitten?)<br>• Korrekturmaßnahmen (Welche Korrekturmaßnahmen wurden gemacht? Haben sie geholfen?)<br>• Begutachtungen von externen Stellen (Wie hat mein Labor bei Begutachtungen von extern, wie z.B. Überwachungsbehörden, abgeschnitten?)<br>• Feedback von Mitarbeitern und Externen<br>• Beschwerden<br>• Wirksamkeit von jeglichen umgesetzten Verbesserungen<br>• Angemessenheit von Ressourcen (Bin ich mit meinen Ressourcen gut aufgestellt? Gab es Probleme? Was benötige ich, um besser voran zu kommen?)<br>• Ergebnisse der Risikoidentifikation (Welche Risiken für meine Forschungen gibt es? Muss ich Maßnahmen ergreifen?)<br>• Resultat der Sicherstellung der Validität der Ergebnisse<br>• Andere relevante Faktoren wie die Überwachungstätigkeiten und Schulungen (Benötigen die Labormitarbeiter spezielle Schulungen? Wie war die Teilnahmequote an angebotenen Schulungen?)
9.5.3	Ergebnisse von Managementbewertung enthalten Entscheidungen und Maßnahmen zu Folgendem:<br>a) Wirksamkeit des Managementsystems und seiner Prozesse;<br>b) Verbesserung der Labortätigkeiten in Bezug auf die Erfüllung der Anforderungen dieses Dokuments;<br>c) Bereitstellung der erforderlichen Ressourcen;<br>d) jeglichem Erfordernis für Änderungen.		

- Leistungsfähigkeit der Lieferanten (Gibt es schlechte Lieferanten, die mein Labor beliefern? Kann ich daran etwas ändern?)

Vorgehen für Dokumentation:
Dokumentation der Ergebnisse und Maßnahmen des Managementreviews

Erfolgt ein **Managementreview**?
Werden die genannten Punkte berücksichtigt?

Erfüllt? ☐

9.6 Verbesserung

	Forderung	Ziel der Forderung	Umsetzung
9.6.1	Das Laboratorium erkennt Chancen zur Verbesserung und setzt die notwendigen Maßnahmen um.	Mitarbeiter des Labors können gute Ideen und andere Sichtweisen haben. Diese Ideen sollten gesammelt und geprüft werden (Arbeit als Team, Steigerung der Mitarbeiterzufriedenheit und damit der Effizienz).<br><br>Externes Feedback ist ebenso wichtig für die kontinuierliche Verbesserung und die Effizienzsteigerung des Labors.<br>Beschwerden an das Labor sind zu prüfen. Dem Beschwerdeführer ist, wenn möglich, das Ergebnis der Prüfung mitzuteilen. Das beweist die Seriosität des Labors.	Chancen zur Verbesserung können durch die Prüfung der operativen Verfahren, allgemeinen Ziele, Peer Review-Ergebnisse, Korrekturmaßnahmen, Managementbewertung, Vorschläge des Personals, Risikobewertung, Datenanalyse, Feedback und Eignungsprüfung erkannt werden.<br>Feedbacks dokumentieren, auswerten, gegebenenfalls Maßnahmen einleiten und archivieren.<br>Wenn möglich: Erhalt der Beschwerde bestätigen, über Fortschritte und das Ergebnis berichten.<br><br>**Vorgehen für Dokumentation:**<br>Erstellen einer Feedbackliste.
9.6.2	**Internes Feedback**<br>Die Mitarbeiter werden ermutigt Empfehlungen zur Verbesserung des Labors abzugeben. Diese werden durch die Leitung aufbewahrt und gegebenenfalls umgesetzt.<br>Feedbacks und Maßnahmen werden dokumentiert.<br>Die feedbackgebenden Mitarbeiter erhalten eine Rückmeldung.		
9.6.3	**Externes Feedback und Beschwerden**<br>Sowohl positive als auch negative Informationen werden dokumentiert. Das Feedback wird ausgewertet und für die Verbesserung des Managementsystems und der Labortätigkeiten genutzt.		

Im Falle von Beschwerden erfolgt ein Verfolgen und Aufzeichnen, einschließlich der Maßnahmen, die zu ihrer Lösung ergriffen werden. Wenn möglich, ist dem Beschwerdeführer der Erhalt der Beschwerde zu bestätigen. Über Fortschritte und das Ergebnis ist zu berichten.	Fehlerhafte Prozesse werden aufgedeckt und verbessert.	Gibt es eine Möglichkeit für **Mitarbeiterfeedback**?<br>Wird Feedback dokumentiert, ausgewertet, gegebenenfalls umgesetzt und eine Rückmeldung an den Mitarbeiter gegeben?<br>**Externes Feedback** dokumentieren und auswerten, gegebenenfalls Maßnahmen umsetzen?<br>Gibt es ein Verfahren für Bewertung und Bearbeitung von **Beschwerden**?<br><br>**Erfüllt? ☐**
